# Topical delivery of a small molecule RUNX1 transcription factor inhibitor for the treatment of proliferative vitreoretinopathy

**DOI:** 10.1038/s41598-020-77254-0

**Published:** 2020-11-30

**Authors:** Santiago Delgado-Tirado, Dhanesh Amarnani, Guannan Zhao, Elizabeth J. Rossin, Dean Eliott, John B. Miller, Whitney A. Greene, Leslie Ramos, Said Arevalo-Alquichire, David Leyton-Cifuentes, Lucia Gonzalez-Buendia, Daniela Isaacs-Bernal, Hannah A. B. Whitmore, Natalia Chmielewska, Brandon V. Duffy, Eric Kim, Heuy-Ching Wang, Jose M. Ruiz-Moreno, Leo A. Kim, Joseph F. Arboleda-Velasquez

**Affiliations:** 1 Schepens Eye Research Institute of Massachusetts Eye and Ear and the Department of Ophthalmology at Harvard Medical School, Boston, USA; 2 Retina Service, Massachusetts Eye and Ear and the Department of Ophthalmology at Harvard Medical School, Boston, USA; 3 Sensory Trauma Task Area, United States Army Institute of Surgical Research, Fort Sam Houston, San Antonio, USA; 4 Energy, Materials and Environment Group, Faculty of Engineering, Universidad de La Sabana, Chia, Colombia; 5 Universidad EIA, Envigado, Colombia; 6 Boston College, Boston, USA; 7 Harvard College, Cambridge, USA; 8 Department of Ophthalmology, Castilla La Mancha University, Puerta de Hierro-Majadahonda University Hospital, Madrid, Spain; 9 Vissum Corporation, Alicante, Spain

**Keywords:** Biotechnology, Cell biology, Drug discovery, Medical research

## Abstract

Proliferative vitreoretinopathy (PVR) is the leading cause of retinal detachment surgery failure. Despite significant advances in vitreoretinal surgery, it still remains without an effective prophylactic or therapeutic medical treatment. After ocular injury or retinal detachment, misplaced retinal cells undergo epithelial to mesenchymal transition (EMT) to form contractile membranes within the eye. We identified Runt-related transcription factor 1 (RUNX1) as a gene highly expressed in surgically-removed human PVR specimens. RUNX1 upregulation was a hallmark of EMT in primary cultures derived from human PVR membranes (C-PVR). The inhibition of RUNX1 reduced proliferation of human C-PVR cells in vitro, and curbed growth of freshly isolated human PVR membranes in an explant assay. We formulated Ro5-3335, a lipophilic small molecule RUNX1 inhibitor, into a nanoemulsion that when administered topically curbed the progression of disease in a novel rabbit model of mild PVR developed using C-PVR cells. Mass spectrometry analysis detected 2.67 ng/mL of Ro5-3335 within the vitreous cavity after treatment. This work shows a critical role for RUNX1 in PVR and supports the feasibility of targeting RUNX1 within the eye for the treatment of an EMT-mediated condition using a topical ophthalmic agent.

## Introduction

Proliferative vitreoretinopathy (PVR) is a condition in which, after retinal detachment or ocular trauma, some retinal cells are displaced from their anatomical location, undergo epithelial to mesenchymal transition (EMT), and grow uncontrollably beneath or on top of the retina triggering the formation of retinal membranes, tractional retinal detachment, and permanent vision loss^1,2^. PVR occurs in 5–10% of all rhegmatogenous retinal detachments and in 40–60% of patients with open globe injuries^3–5^. The current standard of care for the treatment of PVR is pars-plana vitrectomy, an ocular surgical procedure performed to remove tractional membranes^6^. Although anatomical outcomes are satisfactory, final visual acuity is usually poor (40–80% of patients only recover 5/200 of vision)^7^. Various pharmacological agents have been tested for the treatment of PVR targeting inflammation^8–10^, cell proliferation^11–15^, and growth factors^16^; albeit without success^17^. Currently, there are no specific therapeutic agents used for the prevention or treatment of PVR^16,18^.

EMT is a biological mechanism that allows polarized epithelial cells to display phenotypes characteristic of mesenchymal cells including enhanced proliferative and migratory capacity, invasiveness, and increased production of extracellular matrix^19,20^. Snail, Slug, and Twist are transcription factors that respond to extracellular triggers of EMT by executing cellular programs suppressing epithelial-specific proteins including E-cadherin and ZO-1 and inducing mesenchymal-specific proteins including N-cadherin and α-smooth muscle actin (α-SMA)^19,20^. The complexity of the molecular circuitry regulating EMT has limited our ability to develop specific therapeutic interventions to limit pathologic EMT, a process critical to prevalent human conditions including cancer and fibrosis^19,20^.

EMT of retinal pigment epithelial (RPE) cells plays a critical role in the pathobiology of PVR^21–24^. Under physiological conditions, RPE cells form a polarized monolayer underneath the retina that disposes of photoreceptor outer segments via phagocytosis. Upon retinal detachment or trauma, RPE cells are misplaced from their anatomical location and induced to undergo EMT under the stimuli of growth factors, inflammatory cytokines, and exposure to vitreous, a collagenous gel that fills the space between the lens and the retina^22,25^. In fact, RPE readily reproduce critical aspects of PVR in vitro and in vivo under specific stimuli known to trigger EMT including TGF-β2, TNF-α, and vitreous, among others^22,24,26–33^. Other cell types including Müller glial cells and circulating immune cells also infiltrate the retina and contribute to the formation of PVR membranes upon trauma^34–39^. To incorporate the complexity of cell types involved in the pathobiology of PVR, we previously developed primary cultures obtained from human PVR membranes^40^. These primary cultures, which we named C-PVR, proliferate, retain the expression of cell identity markers in culture, and form membranes and band-like structures in culture as found clinically in patients^40^. PVR membranes and C-PVR cultures derived from them represent a readily accessible and unique resource to study human EMT.

Our group previously reported on a critical role of Runt-related transcription factor 1 (RUNX1) in retinal aberrant angiogenesis by analysis of vascular endothelial cells from patients with proliferative diabetic retinopathy (PDR)^41^. With this precedent, we sought to test the hypothesis of whether RUNX1 also plays a role on PVR. RUNX1 is the DNA-binding subunit (or α subunit) of the heterodimeric transcription factor core-binding factor (CBF) that also includes CBFβ, the non-DNA-binding subunit^42^. Binding of CBFβ to RUNX1 enhances RUNX1 binding of DNA initiating regulation of specific transcriptional targets^43^. In different contexts including hematopoiesis, vascular development, and cancer, among others, cells alternate between developmental fates using RUNX1 as a transcriptional switch to control cell proliferation, differentiation, survival, migration and invasion^42,44–48^. One of the most studied functions of RUNX1 relates to the process of endothelial to hematopoietic transition (EHT) where hemogenic endothelial cells become pre-hematopoietic stem and progenitor cells during embryogenesis^49,50^. However, many reports have been also published linking RUNX1 and EMT in non-ocular conditions^46–48,51–53^. Here we present evidence for RUNX1 as a master regulator of cell fates in EMT downstream TGF-β2 in PVR. We also tested the preclinical feasibility of a novel modality of treatment for PVR based on topical application of a small molecule RUNX1 inhibitor as means to limit EMT^54^.

## Results

### RUNX1 expression in human PVR membranes

**Figure 1:**
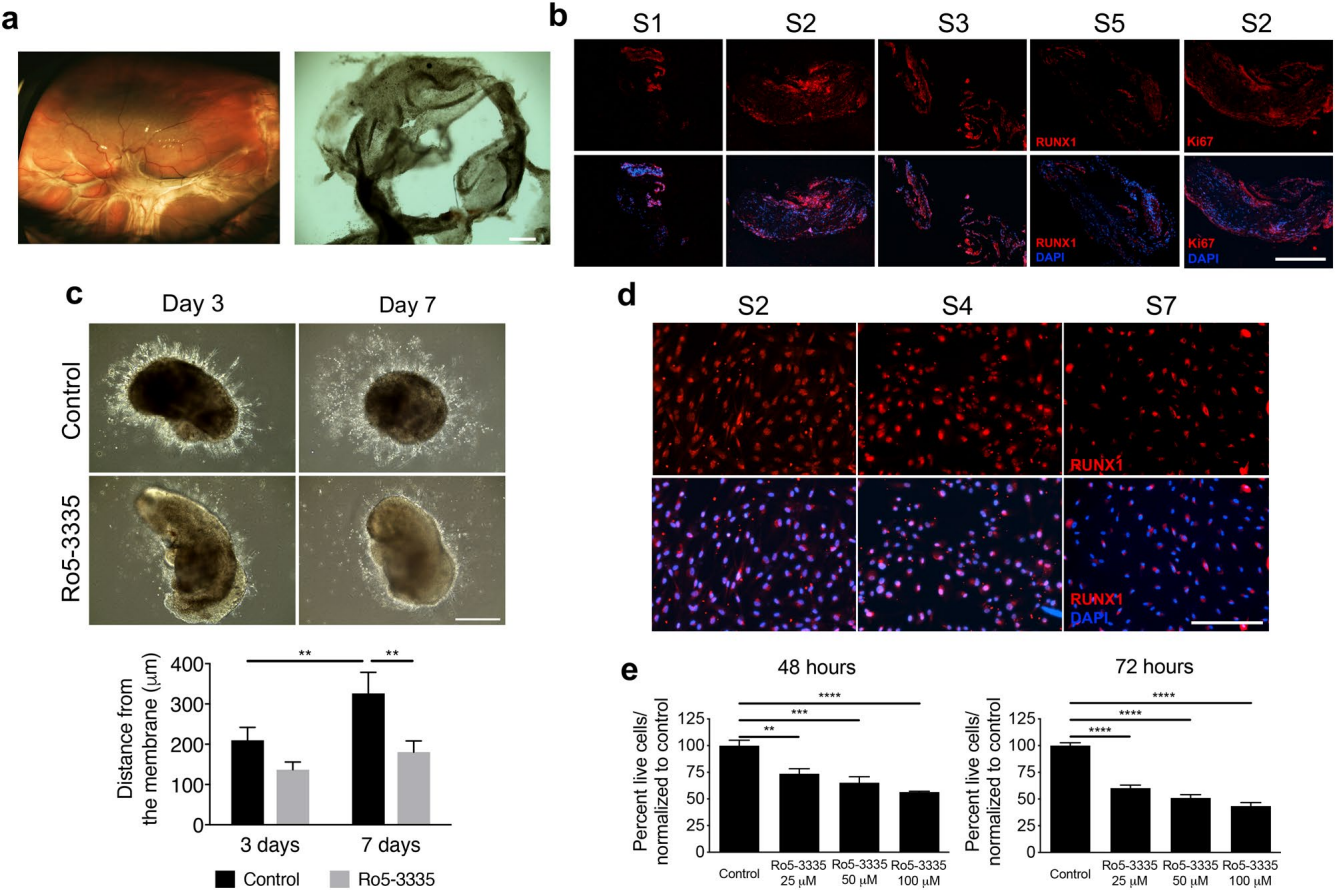
RUNX1 characterization of human PVR membranes. (**a**) Funduscopic image of a patient with PVR. Large preretinal membranes are observed creating retinal folds (left). Representative macroscopic appearance of human specimen obtained during PVR surgery (right). Note high degree of pigmentation denoting the presence of RPE cells within the pathological excised tissue. Scale bar: 400 μm. (**b**) RUNX1 positive cells are clearly identified in human PVR specimens obtained from four different donors. Positive Ki67 labelling was found in those cells with an active proliferative state within the tissue. Scale bar: 400 μm. (**c**) Cell proliferation and sprouting in human PVR explants were evaluated. A reduction in sprouting distance from the specimen is observed 7 days in explants treated with 150 μM Ro5-3335 (**p < 0.001, two-way ANOVA; n = 3 represented as mean ± SEM). Scale bar: 400 μm. (**d**) C-PVR cells from three different donors showed positive staining with RUNX1 antibody. Scale bar: 400 μm. (**e**) RUNX1 inhibition with Ro5-3335 inhibitor reduces C-PVR cells proliferation in vitro in a dose-dependent manner (25 μM, 50 μM and 100 μM), at 48 and 72 h (**p < 0.01, ***p < 0.001, ****p < 0.0001, one-way ANOVA; n = 4 represented as mean ± SEM).

PVR membranes as shown in Fig. 1a, are usually triggered as a consequence of ocular trauma, and unlike PDR, PVR is not driven by aberrant retinal angiogenesis, but instead is considered as an excessive wound-healing process. We found widespread RUNX1 expression in human surgical PVR samples from a total of four donors using immunohistochemistry (Fig. 1b). This was surprising because PVR membranes are devoid of blood vessels and known instead to consist of cells at various stages of the EMT continuum^31,55–57^. RUNX1 expression was identified in areas of cell proliferation identified by Ki67 expression, a marker of active cell division (Fig. 1b).

### RUNX1 inhibition limits PVR proliferation ex-vivo

We developed an ex-vivo model of PVR by growing fragments of freshly isolated human PVR membranes in Matrigel to examine a potential role for RUNX1 in growth of PVR membranes. Phase contrast microscopy of Matrigel-embedded untreated control samples showed long and robust outgrowths from a freshly isolated human PVR membrane over a period of 3 to 7 days (219.9 ± 31.7 μm and 326.2 ± 52.1 μm). In contrast, we observed almost no outgrowths in samples treated with Ro5-3335 (150 μM), a small molecule RUNX1 inhibitor^54^ (Fig. 1c), and the few outgrowths present were significantly shorter after 7 days (136.9 ± 18.9 μm and 180.3 ± 27.9 μm) (**p < 0.01). This finding was confirmed in PVR membranes from two additional donors suggesting a consistent link between RUNX1 function and PVR membrane growth.

### RUNX1 regulates proliferation and TGF-β2-induced EMT in C-PVR

**Figure 2:**
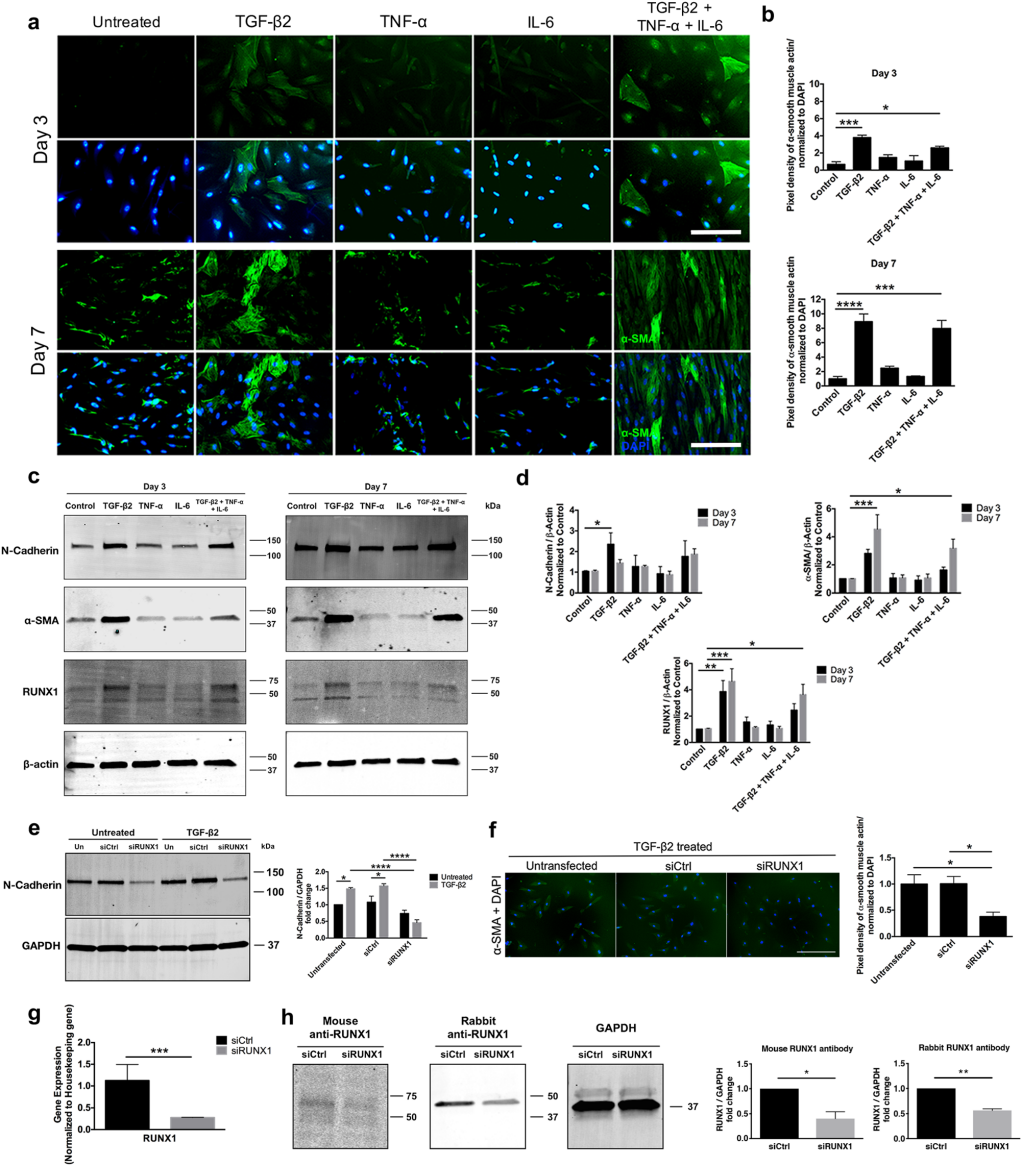
Growth factor-induced EMT in C-PVR cells show upregulation of EMT markers. (**a**) After stimulation with TGF-β2 or combination treatment (TGF-β2 + TNF-α + IL-6), significant changes in α-SMA staining were identified at day 3. These changes in α-SMA expression were more prominent 7 days after induction. (**b**) Increased α-SMA expression levels were detected after stimulation with TGF-β2 and combination when compared to control at day 3 (*p < 0.05, ***p < 0.001, one-way ANOVA; n = 6 represented as mean ± SEM) and day 7 (***p < 0.001, ****p < 0.0001, one-way ANOVA; n = 6 represented as mean ± SEM). (**c**) TGF-β2 and combination treated cells showed increased protein expression of mesenchymal markers (α-SMA and N-Cadherin) at days 3 and 7 after induction. RUNX1 protein levels were also upregulated after induction by TGF-β2 and combination treatment. (**d**) An increase in N-Cadherin was observed at day 3 and in α-SMA protein levels at day 3 and day 7 after induction by TGF-β2 and combination treatments (*p < 0.05, ***p < 0.001, two-way ANOVA; n = 3 represented as mean ± SEM). Similarly, increased protein expression of RUNX1 was observed at day 3 and day 7 when C-PVR cells were stimulated with TGF-β2, and at day 7 with the combination of growth factors (*p < 0.05, **p < 0.01, ***p < 0.001, two-way ANOVA; n = 3 represented as mean ± SEM). (**e**) N-Cadherin expression is reduced by RUNX1 knockdown via siRUNX1 in untreated and TGF-β2-induced cells (*p < 0.05, ****p < 0.0001, two-way ANOVA; n = 3 represented as mean ± SEM). (**f**) TGF-β2-induced α-SMA expression is reduced by RUNX1 knockdown by siRUNX1 (*p < 0.05, one-way ANOVA; n = 12 represented as mean ± SEM). (**g**) siRUNX1 induced a 70% reduction of RUNX1 expression measured by qRT-PCR 48 h post-transfection (***p < 0.001, two-tailed unpaired T-test; n = 3 represented as mean ± SEM). (**h**) Validation of siRUNX1 effect on RUNX1 using mouse and rabbit anti-RUNX1 antibodies. Protein levels quantification of RUNX1 showed 60% and 50% reduction of RUNX1 (*p < 0.05, **p < 0.01, two-tailed unpaired T-test; n = 2 represented as mean ± SEM). Samples used for quantitative comparisons derive from the same experiment and blots were processed in parallel. Representative immunoblots showing cropped images of the same gel for separation of markers. Full-length blots/gels are presented in Supplementary Figures S7–S9.

We analyzed the expression of RUNX1 in primary cultures from PVR membranes (C-PVR) obtained from three donors generated using our previously published protocols to determine whether RUNX1 expression was retained upon cell culture. We confirmed robust RUNX1 expression in these proliferating cultures under basal conditions in approximately 99% of cells from all donors (Fig. 1d). Inhibition of RUNX1 function using Ro5-3335^54^, strongly inhibited the proliferation of C-PVR in a dose-dependent manner, as measured by the CyQUANT Direct Cell Proliferation Assay (Fig. 1e), suggesting a functional role for RUNX1 activity in PVR. Very low levels of cell death were detected by lactate dehydrogenase (LDH) analysis suggesting a direct effect in proliferation by RUNX1 inhibition (see Supplementary Fig. S1). We sought to establish an in vitro model of EMT using our C-PVR cultures to further examine a potential functional role of RUNX1 in PVR. We found that TGF-β2 treatment strongly induced the expression of EMT markers including α-SMA and N-cadherin in C-PVR using immunofluorescence (Fig. 2a,b) and Western blot analyses (Fig. 2c,d). RUNX1 expression was also strongly induced by TGF-β2 treatment (Fig. 2c,d). These effects were detected at 3-days after treatment and significantly increased over time by day 7. TNF-α or IL-6 treatments failed to induce EMT markers or RUNX1 expression and the combination treatment with all three growth factors did not appear to be more effective than TGF-β2 alone suggesting lack of additive or synergistic effects.

We used siRNA to knockdown RUNX1 expression in C-PVR to determine whether RUNX1 expression was necessary for TGF-β2-induced EMT. We found that RUNX1 knockdown effectively blunted the response of C-PVR to TGF-β2 treatment by preventing morphological changes associated with EMT and by reducing N-Cadherin expression (Fig. 2e). A significant reduction in the induction of the EMT marker α-SMA by TGF-β2 treatment was also observed using immunofluorescence (Fig. 2f). Under our experimental conditions siRNA effectively reduced RUNX1 expression by 70% as determined by qRT-PCR (Fig. 2g). Downregulation of RUNX1 via siRNA was validated by Western blot analysis using mouse anti-RUNX1 and rabbit anti-RUNX1 antibodies (Fig. 2h).

### Development of a rabbit model of PVR using human C-PVR cells

**Figure 3:**
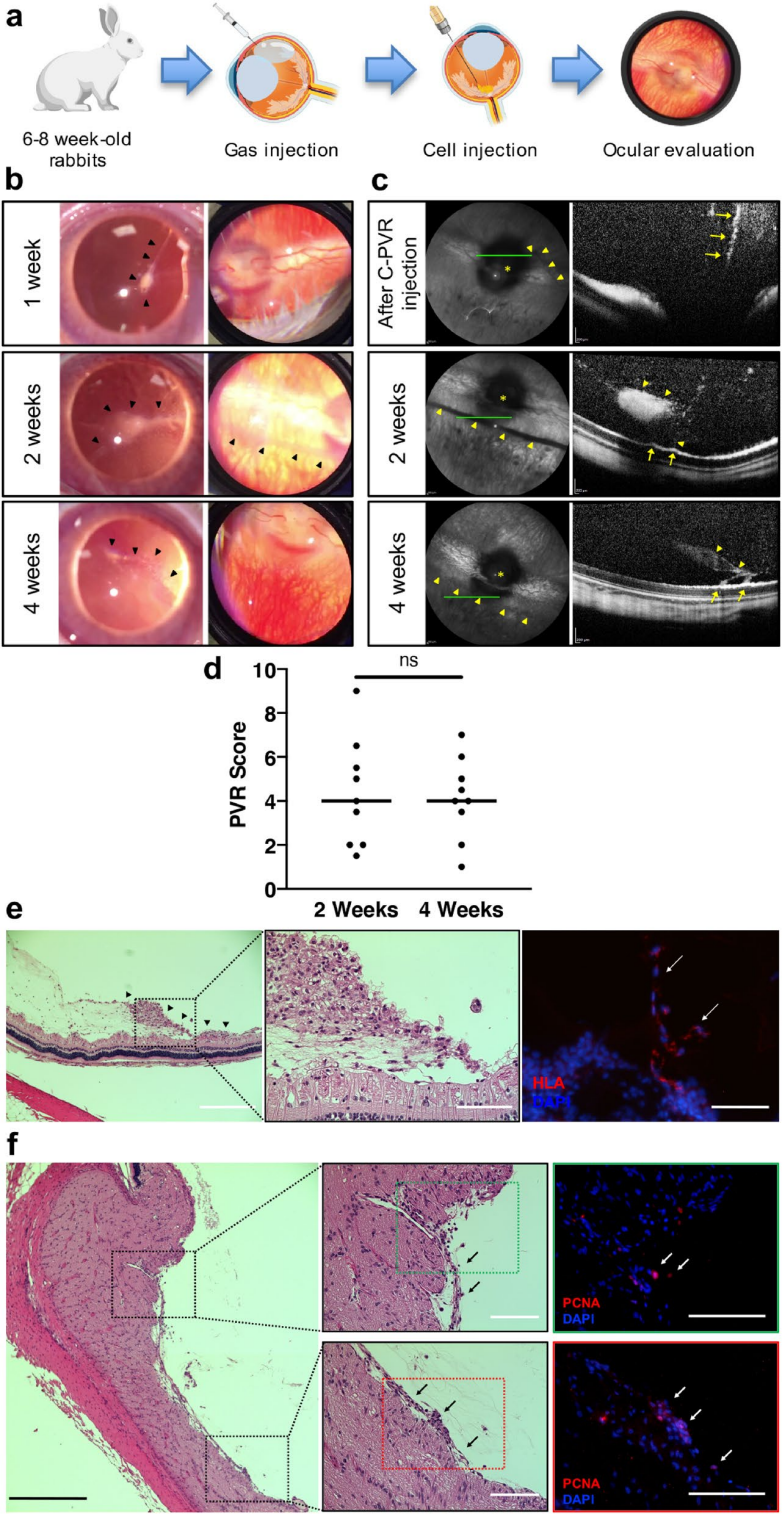
Development of a new PVR model using C-PVR cells. (**a**) Schematic representation of experimental design. Created with BioRender.com. (**b**) Representative images of PVR-like findings. Right eye of the same animal of study is displayed. After 1 week of follow up, vitreous floaters due to cell proliferation are easily identified with no changes in fundus (arrowheads). At 2 weeks, large intravitreal membranes and floaters within the vitreous body are noted (arrowheads). In the fundus image, epiretinal membrane (ERM) is identified (arrowheads). Also, reduced visualization of posterior pole due to vitreous haze is noted. However, at 4 weeks, cell proliferation has decreased, and the size of intravitreal membranes and ERMs is reduced (arrowheads). (**c**) Immediately after cell injection, funduscopy shows a cluster of cells that can be readily identified in the vicinity of the optic nerve (arrowheads) and cells are visible over optic nerve area by OCT (arrows). After 2 weeks, injected cells have proliferated and formed an ERM (arrowheads). By OCT a thick ERM (arrowheads) causing focal traction over retinal surface can be seen (arrows). After 4 weeks, membranes and vitreous strands are still visible (arrowheads), but show decreased traction of retinal surface (arrows). (asterisk: optic nerve head, green line depicts OCT scan area). (**d**) PVR Score results at 2 and 4 weeks of follow-up. Data suggest lack of progression from the second to the fourth week of the evaluation. Line is expressing the median of each studied group. (**e**) Histologic characterization of C-PVR injected eyes 4 weeks after cell injection. Presence of ERM can be observed over retinal surface area (arrowheads). (H&E, Scale bar: 400 μm). Dotted lines: detailed area of ERM causing retinal focal traction. (H&E, Scale bar: 100 μm). Anti-HLA antibody was used to confirm presence of human cells inside injected rabbit eyes (arrows). (Scale bar: 400 μm). (**f**) ERMs could be identified over the area of the optic nerve and medullary ray (Scale bar: 400 μm). Magnified areas signifying locations where ERMs were seen (arrows) (Scale bar: 100 μm). PCNA positive cells are observed within the ERMs formed (Scale bar: 100 μm).

We injected 1 × 10^6^ C-PVR cells per eye inside the vitreous cavity in nine adult rabbits to develop a novel model of PVR using human PVR primary cultures (Fig. 3a). Placement of the cells over the optic nerve area was confirmed in live rabbits using fundus imaging and optical coherence tomography (OCT) immediately after cell injection. We evaluated the progression of the disease one-week after injection and performed indirect ophthalmoscopy, fundus, and OCT imaging at two- and four-weeks. Rabbits were euthanized prior to tissue collection after imaging at the 4-week time point.

We observed robust growth of intravitreal and epiretinal membranes at the 2-week time point in all animals. In 3 out of 9 rabbits these membranes caused focal traction of the retina, a hallmark of PVR. Most common findings identified were the formation of vitreous floaters, vitreous strands, intravitreal and epiretinal membranes with or without retinal traction, which were seen at 2- and 4-weeks (Fig. 3b,c). We developed a scoring system using a combination of clinical and imaging characteristics of PVR including presence of vitreous floaters, membranes, and traction of retinal tissue to grade PVR severity (see Supplementary Table S1). Inclusion of imaging features is important because prior grading systems, such as the Fastenberg scale, traditionally used to grade PVR, only assessed clinical criteria as it was developed before OCT became widely available^58^. Using our PVR score system, we observed a progression of the phenotype from the time of injection to the 2-week time point, but we observed no additional progression beyond this time point after 4 weeks of follow-up (Fig. 3d). Therefore, we used the two-weeks time point for preclinical efficacy experiments because there was no significant change afterwards. A high degree of reliability was found between graders when performing PVR Score measurements. The average intra-class coefficient was 0.976 with a 95% confidence interval from 0.957 to 0.986. Histological analyses of whole eye sections showed PVR-like findings that accurately correlated with the live imaging observations including intravitreal and epiretinal membranes with or without traction (Fig. 3e). We also demonstrated that PVR-like membranous tissue inside the rabbit eye was of human origin using human anti-HLA antibody (Fig. 3e). Detection of Proliferating Cell Nuclear Antigen (PCNA) positive staining confirmed the presence of proliferating cells within pathological tissues (Fig. 3f). Fibronectin staining, a marker of extracellular matrix deposition, was also identified in experimental PVR membranes (see Supplementary Fig. S2).

### Development of a nanoemulsion formulation of a RUNX1 inhibitor for topical ophthalmic administration

**Figure 4:**
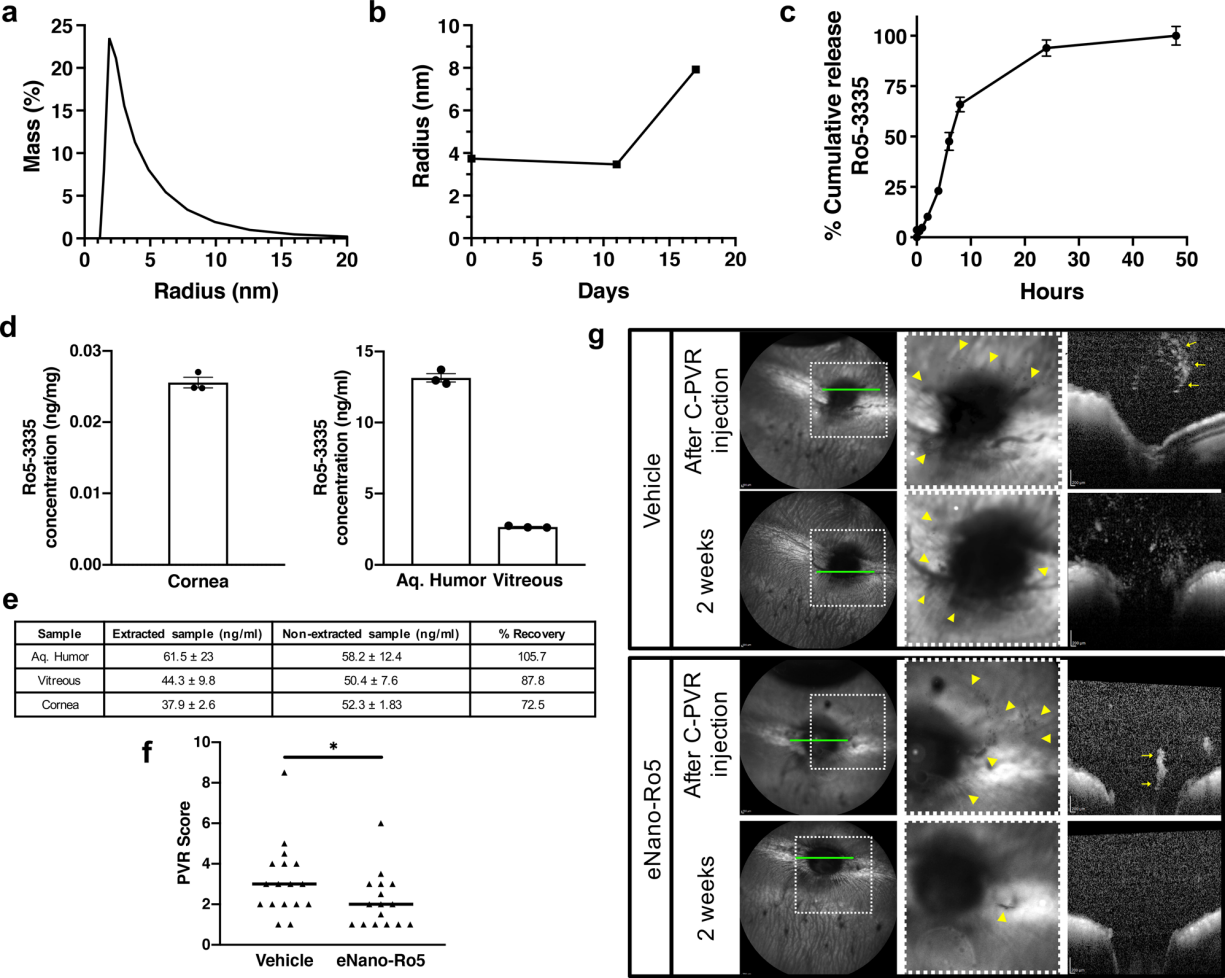
Ocular topical application of nanoemulsion (eNano-Ro5) reduces PVR severity using a new rabbit model of PVR. (**a**) Unimodal distribution of drop size within the nanoemulsion. (**b**) Time-course characterization of drop radius. Stability of the nanodrop size in the formulated emulsion 17 days after production is shown. (**c**) Release profile of small molecule Ro5-3335 from eNano-Ro5. Results are expressed as mean ± SEM for n = 3. (**d**) Distribution of Ro5-3335 in rabbit cornea, aqueous humor and vitreous after 28 days of treatment with eNano-Ro5 3 times a day. Results are expressed as mean ± SEM, n = 3. (**e**) Detection of Ro5-3335 in normal rabbit ocular tissues. Comparison between non-extracted and extracted samples at a concentration of 50 μg/mL. (**f**) Quantification of PVR severity within both study groups adhering to PVR Score grading system. A reduction in PVR Score was found in the group treated with eNano-Ro5 (n = 16) compared to vehicle (n = 17) (*p < 0.05, two-tailed Mann–Whitney test). The line is denoting the median of each studied group. (**g**) Representative imaging results in both groups. In the vehicle-treated group, multiple cell clumps and deposits can be identified after cell injection (arrowheads). These findings are also observed by OCT (arrows). Increased cell density was observed after 2 weeks of treatment with vehicle. A reduction in cellular density and progression was observed after 2 weeks of treatment with eNano-Ro5 by fundus imaging (arrowheads) and OCT (arrows). Green line depicts OCT scan area, dotted lines represent a detailed area of posterior pole images.

To test the preclinical efficacy of RUNX1 inhibition using Ro5-3335 in our newly developed rabbit model, we developed a formulation ideally suited for topical ophthalmic administration using eye drops. Ro5-3335 is a highly lipophilic small molecule, thus, we designed a nanoemulsion formulation utilizing surfactants as the encapsulation matrix within an aqueous phase. We used lecithin and isopropyl myristate, as the oil phase, non-toxic matrices which have been previously used in the eye^59^. For the aqueous phase we used phosphate buffered saline (PBS). Analysis of the nanoemulsion, which we called eNano-Ro5, using dynamic light scattering (DLS) showed that our formulation resulted in particles with an average radius of 4 nm with a unimodal distribution (Fig. 4a). The nanoemulsion remained stable in size for at least 11 days increasing to only about 8 nm in size by 17 days (Fig. 4b). We also investigated the release profile of Ro5-3335 from eNano-Ro5. Ro5-3335 released from the nanoemulsion was sustained for 24 h in vitro, a time at which a rapid equilibrium was reached. (Fig. 4c). We confirmed penetration of topical application of eNano-Ro5 within rabbit ocular structures by liquid chromatography-tandem mass spectrometry (LC–MS/MS). Using 100 μL of 7.92 mM eNano-Ro5 administered three times a day over 4 weeks, detectable levels of Ro5-3335 were found in the cornea (0.03 ng/mg), aqueous humor (13.15 ng/mL) and vitreous (2.67 ng/mL) (Fig. 4d) (for additional data at a 2-week time point see Supplementary Fig. S3.) Liquid–liquid microextraction efficiency recovery values are shown in Fig. 4e. Furthermore, topical ocular administration of eNano-Ro5 was safe and well tolerated as no significant anterior segment abnormalities suggestive of ocular toxicity were found following Semiquantitative Preclinical Ocular Toxicology Scoring (SPOTS) guidelines (see Supplementary Fig. S4a,c) after 2 or 4 weeks of treatment. We did not observed changes indicating cell death in the posterior segment assessed via terminal deoxynucleotidyl transferase dUTP nick end labeling (TUNEL) and H&E (see Supplementary Fig. S5 and S6, respectively). No significant differences in intraocular pressure (IOP) levels were observed throughout the study (see Supplementary Fig. S4b). A complementary safety analysis in mice showed no alteration in electroretinography (ERG) values after 1 week of treatment with topical eNano-Ro5 (see Supplementary Fig. S4d).

### RUNX1 inhibition reduces PVR in a rabbit model

We tested the preclinical efficacy of Ro5-3335 to reduce progression of PVR by treating rabbits starting immediately after cell injection with 100 μL of eNano-Ro5 or vehicle, applied as eye drops on the ocular surface three times a day. We found that rabbits treated with eNano-Ro5 had a significant reduction of PVR severity after 14 days of follow-up compared to vehicle-treated controls (*p < 0.05). Animals treated with eNano-Ro5 had a median PVR score of 2 (n = 16) versus animals treated with the vehicle control, which had a median PVR score of 3 (n = 17) (Fig. 4f,g). Due to feasibility we were not able to include side-by-side comparisons of untreated control rabbits therefore an effect of the vehicle alone cannot be assessed.

## Discussion

PVR is the leading cause of retinal detachment surgery failure and to date, despite significant advances in vitreoretinal surgery, it remains without an effective prophylactic or therapeutic medical treatment^60^. In this study, we identified RUNX1 as a mediator of EMT in PVR. Using our previously developed primary culture model of PVR and a newly developed PVR in vivo model, we successfully inhibited EMT and PVR progression via RUNX1 inhibition. To our knowledge this is the first report of successful preclinical modulation of a transcription factor to improve outcomes of an ocular condition driven by EMT^46–48,50–53^.

RUNX1 was detected in a subpopulation of cells that are in a highly proliferative stage in human PVR surgical specimens. Histopathology confirmed that RUNX1 expression in PVR was more widespread than the vascular pattern we previously reported for RUNX1 in PDR membranes^41^. Additionally, C-PVR cells also displayed high levels of RUNX1 expression in vitro. When these cells were stimulated by growth factors known to promote EMT (TGF-β2), RUNX1 expression levels were significantly increased along with mesenchymal markers including N-Cadherin and α-SMA. This response was effectively prevented by RUNX1 knockdown suggesting a mechanistic link between RUNX1 expression and EMT in PVR.

We generated a new rabbit model of PVR using injection of human primary cultures derived from PVR membranes in an attempt to reproduce some of the complexity of the disease. Rabbit is commonly used to model PVR^21,33^. Ocular anatomic characteristics such as a large globe, vitreous cavity, and relatively small lens support the use of rabbits as a suitable animal model to study PVR^21,33^. Importantly, rabbits are widely used in research to test pharmacologic efficacy, predictability, ocular safety and tolerability of new drugs and drug delivery devices due to anatomic and physiological similarities with human eyes^61,62^. Previous animal models of PVR have used surgical manipulation or injection of dermal fibroblasts or transformed cell lines to identify candidate treatments but to date these have not translated into effective therapies^21,39,60^.

Using our rabbit model, we showed that topical application of a nanoemulsion containing a RUNX1 inhibitor effectively reduced progression of PVR. This suggests that RUNX1 modulation could be a novel therapeutic strategy for complications of retinal detachment and ocular trauma. Unlike other published models, in our approach we injected C-PVR cells intravitreally but avoided surgical manipulation such as lens extraction, induction of retinal breaks or injection of blood or plasma. As a result, we obtained a mild phenotype that does not cause retinal detachment. We reasoned that our system is therefore useful to model early stages of the disease were migration and proliferation of cells is occurring and pharmacological treatments could help. This modality of treatment would be suitable in patients to prevent the onset or progression of PVR before and/or after surgical repair of retinal detachment or ocular trauma, when the disease is mild (i.e. early stages), and medical treatments are still a therapeutic option. PVR animal models that result in a very severe phenotype including retinal detachment with massive retinal traction and retinal tears may not be suitable to practically assess the preclinical efficacy of a pharmacological treatment because, much like in humans, the treatment for these robust phenotypes is surgery.

The use of a noninvasive topical ophthalmic formulation instead of intravitreal injections may offer advantages for prolonged drug administration in situations with limited access to medical facilities or highly trained clinicians or under social distancing conditions, as they can be self-administered. Intravitreal drug injections, commonly used for the treatment of vitreoretinal diseases, are invasive and may carry serious risks including vitreous hemorrhage, retinal detachment, central retinal artery occlusion secondary to an acute increase of intraocular pressure, and endophthalmitis^63–66^. In addition, albeit very effective, intravitreal injections of therapeutic agents are commonly administered in a monthly regime, impacting patient quality of life^67^. In the context of PVR, dislodgement of RPE cells and invasion of immune cells starts immediately after retinal or ocular trauma. Therefore, preventing PVR as soon as feasible with an efficacious drug could potentially have significant clinical impact. Thus, there is a clinical need for alternative, less invasive drug delivery methods that could be easily administered to temporize the eye and mitigate damage from this devastating disease.

This study has some limitations. First, there is a need to elucidate the precise mechanisms of how Ro5-3335 affects RUNX1 transcriptional activity and its downstream targets in PVR. Secondly, in developing our rabbit model we avoided surgical manipulation such as lens extraction, induction of retinal breaks or injection of blood or plasma, and merely injected C-PVR cells intravitreally. As a result, we obtained a mild phenotype and further research using models with more severe disease may be warranted. Third, because of feasibility we examined safety of our treatment via ERG in mice after 1 week of treatment. Longer exposures and ERG testing in other animal models need to be considered for future experiments.

EMT is a common process involved in multiple ocular diseases including corneal fibrosis, glaucoma, PVR, PDR and age-related macular degeneration. Hence, the link between RUNX1 and EMT identified in this research could be of relevance in the understating of the pathogenesis of these diseases. In addition, the development of drugs targeting RUNX1 may have multiple applications in the treatment of ocular conditions leading to blindness since RUNX1 also plays a critical role in aberrant ocular angiogenesis^41^.

## Materials and methods

### Sample size

Total animal numbers needed to perform this study were calculated accordingly with the number of animals used in prior in vivo studies, and by sample size power calculation using data from a pilot experiment which was adjusted for non-parametric distribution to detect a difference between the means of 35% with 80% power (and α = 0.05). These numbers represent the number of animal needed to achieve statistical significance and experimental reproducibility. Specifically, the sample size was calculated by using a formula established for parametric variables and then correcting for the non-parametric condition as previously described (Erich L. Lehmann, Nonparametrics: Statistical Methods Based on Ranks, Revised, 1998).

### Randomization

For the purpose of specimens analyzed for immunofluorescence, in vitro and ex vivo experiments, patients undergoing surgery for repair of retinal detachment and removal of PVR membranes were randomly chosen. An ex vivo preliminary experiment was done to determine the time points in which the peak of the outgrowths was consistent throughout the samples. For the proliferation assays, time points were selected to represent the observed growth rate of our cultures and doses were chosen to be consistent with previously reported IC50 data. For in vitro growth factor EMT-induction assays, time points were based on the characteristic phenotype of the cells and on what has been shown in previous studies. In vivo data analysis was performed individually in a randomized and masked manner by two experienced physician observers trained in clinical ophthalmology. An average of both evaluations was obtained.

### Data exclusion criteria

Animals with presence of subretinal, preretinal or vitreous hemorrhage after PVR induction procedure were excluded of the study.

### Replicates

All the in vitro experiments were conducted using three biological repeats and were performed in three independent experiments. For the preclinical safety and efficacy evaluation in vivo, three independent experiments were performed and data shown correspond to pooled results.

### Equipment and settings for immunoblots

All the images were acquired and quantified using the Image Studio version 2.1 (LI-COR Biosciences, Lincoln, NE). All the blots were scanned at an intensity of 4.0. The brightness was adjusted uniformly across the entire blot using the Image studio software to visualize the bands for each experiment. Original complete blots are shown in supplementary data and citations for each antibody provided in the methods section.

### Study population

This study was performed at the Schepens Eye Research Institute of Massachusetts Eye and Ear, and research protocols were approved by the Institutional Review Board at Massachusetts Eye and Ear for the collection of surgical specimens and for the retrospective analysis of clinical data. All research protocols adhered to the tenets of the Declaration of Helsinki, signed informed consent form and Health Information Portability and Accountability Act authorization were obtained from all the participants included in the study.

Nine patients were recruited from Massachusetts Eye and Ear who had grade C PVR and were undergoing PVR surgery. The demographics of the patients are summarized in Table 1. Patients had to be at least 18 years old and could not be pregnant. Immediately after surgical extraction, PVR membranes were placed in a specimen cup containing calcium-free and magnesium-free balanced salt solution (BSS) for transportation at room temperature (RT). Within 1–2 h after extraction, membranes were processed under sterile biosafety level 2 conditions for immunofluorescence, cell culture or ex-vivo membrane culture. Biospecimen reporting for improved study quality (BRISQ) reporting guidelines for human biospecimens were followed to complete this section^68^.

**Table 1:** Clinical demographics.

Patient ID	Age (years)	Sex	Cause of retinal detachment	Type of retinal detachment	Class of PVR	Preoperative visual acuity	Postoperative visual acuity	Sample analysis
PVR-02	76	Female	Traumatic, open globe injury zone 3	Recurrent macula-off rhegmatogenous retinal detachment	C	Hand motions	Counting fingers	Fixed (S1)
PVR-03	32	Female	Spontaneous	Recurrent macula-off rhegmatogenous retinal detachment	C	Hand motions	20/150	Fixed and cultured (S2)
PVR-04	67	Female	Spontaneous	Recurrent macula-off rhegmatogenous retinal detachment	C	Hand Motions	20/125	Fixed (S3)
PVR-05	68	Male	Spontaneous	Recurrent macula-off rhegmatogenous retinal detachment	C	Hand motions	Hand motions	Cultured (S4)
PVR-12	64	Female	Traumatic, open globe injury zone 2	Recurrent macula-off rhegmatogenous retinal detachment	C	Hand motions	Hand motions	1st explant
PVR-13	68	Female	Traumatic, open globe injury zone 3	Recurrent macula-off rhegmatogenous retinal detachment	C	Light perception	Hand motions	Fixed (S5)
PVR-14	64	Male	Spontaneous	Recurrent macula-off rhegmatogenous retinal detachment	C	20/600	20/1000	Cultured (S7)
PVR-18	71	Female	Spontaneous	Recurrent macula-off rhegmatogenous retinal detachment	C	Hand Motions	Counting Fingers	2nd explant
PVR-20	24	Female	Spontaneous	Recurrent macula-off rhegmatogenous retinal detachment	C	Light Perception	Counting Fingers	3rd explant

### Immunofluorescence analysis of PVR membranes

Human PVR membranes were fixed and processed for analysis using our previously published protocol^40^. Sections from 4 different human donors were processed for immunofluorescence using the following antibodies: anti-RUNX1 (1:100; LS-B13948; Lifespan Biosciences, Seattle, WA), and anti-human Ki67 antibody (1:100; NB500-170; Novus Biologicals, Ontario, Canada). For heat-induced antigen retrieval the slides were boiled in 10 mM sodium citrate buffer (pH 6.0) and then maintained at a sub-boiling temperature (95–100 °C) for 20 min and subsequently cooled on the bench top for 30 min. Slides were washed with distilled water and permeabilized with 0.5% Triton X-100 in PBS for 5 min and blocked (10% goat serum in PBS) for 1 h at RT. The primary antibody was prepared in antibody dilution buffer (5% goat serum) and samples were incubated overnight with the antibody solution at 4 °C. Membranes were washed with PBS and incubated with goat anti-rabbit Alexa Fluor 594 secondary antibody (1:300; A-11012; Invitrogen, Carlsbad, CA) for 2 h at RT. Slides were mounted and visualized using Prolong Gold Antifade Reagent with DAPI (P36935, Invitrogen). Images were obtained using an EVOS FL automated stage live cell imaging system (Life Technologies, Cambridge, MA).

### C-PVR culture and growth factor induction

Immediately after surgery, the human PVR membranes were processed for isolation and cell culture (C-PVR) using our previously established protocol^40^. C-PVR cells (30 × 10^3^/well) were seeded in 48-well plates for a period of 24–72 h. Then, they were washed with PBS and treated with PVR media alone (control) or in PVR media with TGF-β2, TNF-α, IL-6 or a combination of all three growth factors, using triplicates for each condition. We incubated our C-PVR cells with these growth factors, which have been previously used by others to induce EMT in vitro^19,47,69^.

### Immunocytochemistry and assessment of EMT using immunofluorescence

Our previously established protocol for immunocytochemistry was used to detect the presence of RUNX1 in culture cells from 3 human donors^40^. Cells were fixed with 4% PFA for 10 min, washed with PBS, permeabilized with 0.5% Triton X-100 in PBS for 5 min, and blocked (10% goat serum in PBS) for 1 h at RT. The cultures were incubated with primary antibody rabbit anti-RUNX1, (1:100; LS-B13948, LifeSpan BioSciences) overnight at 4 °C followed by incubation with goat anti-rabbit Alexa Fluor 594 secondary antibody (1:300; A-11012, Invitrogen). Following induction with the growth factors cells were incubated with the primary antibody mouse anti-α smooth muscle actin (1:250; M0851, Dako, Glostrup, Denmark) overnight at 4 °C followed by incubation with goat anti-mouse Alexa Fluor 488 secondary antibody (1:300; A-11001, Invitrogen) for 2 h at RT. Cell nuclei were labeled with Hoechst 33342 diluted in PBS (1:200, 639, ImmunoChemistry Technologies, Bloomington, MN) and counted for quantification using ImageJ software (National Institutes of Health, Bethesda, MD) using our previously established methods protocol^40^. Cells were washed with PBS and images were obtained using an EVOS FL automated stage live cell imaging system (Life Technologies).

### Western blot analysis

Protein concentrations were measured, and equal concentrations of protein were separated using 4–20% SDS-PAGE (456–1094, Bio-Rad Laboratories, Hercules, CA), transferred to polyvinylidene difluoride membranes (Millipore Sigma, Darmstadt, Germany) and blocked using Odyssey Blocking Buffer (LI-COR Biosciences) for 1 h at RT^50^. The membranes were incubated overnight at 4 °C with primary antibodies mouse anti-RUNX1^70^ and mouse anti-N-Cadherin^71^ (Santa Cruz Biotechnology Inc., Dallas, TX), rabbit anti-β-actin^72^ (Cell Signaling Technology, Danvers, MA), and mouse anti-α smooth muscle actin^73^ (Sigma, Natick, MA). GAPDH (Santa Cruz Biotechnology Inc.) was used as loading control for siRNA and RUNX1 antibody validation experiments. After washing, the membranes were probed with IRDye 680RD donkey anti-rabbit, and IRDye 800CW donkey anti-mouse (LI-COR Biosciences) antibodies for 1 h at RT. Immunoreactive bands were visualized using the Odyssey Infrared Imaging System, and band intensities normalized to β-actin or GAPDH were quantified using Image Studio (LI-COR Biosciences) using our previously developed protocol^50^. RUNX1 antibody validation control was performed as shown in Fig. 2h. Full-length blots/gels are presented in supplementary information (see Supplementary Figs. S7–S9). Processing of the blots was performed only to adjust the brightness using Acorn software (Flying Meat Inc., Seattle, WA).

### RUNX1 inhibition in an ex vivo model of PVR using Ro5-3335

PVR membranes were divided into pieces and embedded in growth factor reduced Matrigel (354,230; BD Biosciences, San Jose, CA) in a 24-well plate and placed at 37 °C for 30 min for the Matrigel to solidify. The specimens were treated with either vehicle or 150 μM of a small molecule inhibitor of RUNX1 (Ro5-3335; 219506, Millipore Sigma, Darmstadt, Germany) in 500 μl in PVR growth media. Phase contrast images using an EVOS FL imaging system (Life Technologies) were taken and the distance of growth from the embedded tissue was quantified using Image J (National Institutes of Health).

### Small Interfering RNA gene knockdown

Small interfering RNA (siRNA) (75 nmol/L) (Integrated DNA Technologies; CCUUUCAUGUUAAUCAAACAAGUGA, UCACUUGUUUGAUUAACAUGAAAGGGA) sequences were transfected for 12 h using DharmaFECT 1 (GE Life Sciences/Dharmacon, Lafayette, CO) in Opti-MEM (Life Technologies) and incubated in PVR medium with 2% FBS. The culture medium was switched to complete PVR growth media after 12 h.

### Induction of experimental PVR in rabbits

This study was approved by Institutional Animal Care and Use Committee (IACUC) of the Massachusetts Eye and Ear Infirmary. All animal experiments were performed in accordance with the guidelines for the Use of Animals in Ophthalmic and Vision Research of the Association for Research in Vision and Ophthalmology (ARVO). Information regarding in vivo experiments reported in this manuscript are in adherence with the ARRIVE guidelines^74^. Male and female New Zealand White rabbits (2.3 kg of weight, 6–8 week-old) were purchased from Charles River (Charles River Laboratories, Inc., Wilmington, MA). Animals were maintained in a temperature-controlled, 12-h day-night cycle environment with food and water ad libitum. General welfare assessment was performed before surgery and general clinical monitoring was performed daily for the first week, and then every three days after C-PVR injection until completion of the experiment. Before every procedure, animals were anesthetized by intramuscular injection of Ketamine (30–50 mg/Kg) (KetaVed, Vedco Inc., St. Joseph, MO), Xylazine (5–10 mg/Kg) (Anased, Akorn Animal Health, Lake Forest, IL) and Acepromazine (0.75 mg/Kg) (Phoenix Pharmaceuticals Inc., Burlingame, CA). Also, a subcutaneous injection of Buprenorphine (0.05–0.1 mg/Kg) (Buprenex, Reckitt Benckiser Inc., Richmond, VA) was performed. Pupils were dilated with topical application of 1% Tropicamide drops (Bausch and Lomb Inc., Tampa, FL), anesthetic drops of 0.5% Proparacaine (Bausch and Lomb Inc.) were also applied. Oxygen levels and heart rate were continuously monitored until complete anesthesia recovery. Right eye of each animal was used to develop the model and left eyes were used as controls. To develop our model, we first generated a master stock of C-PVR cells from a donor to be used at a similar passage for intravitreal injections to maintain uniformity of the system between experiments. Gas displacement of the vitreous was induced by intravitreal injection of 0.15 mL of perfluoropropane (C_3_F_8_) (Alcon, Fort Worth, FL) 3 mm behind the limbus. Three days after gas displacement, intraocular pressure (IOP) was reduced by gas withdrawal. Subsequently, 0.2 mL of BSS containing approximately 1 × 10^6^ C-PVR cells were intravitreally injected. These cells were obtained and processed following our previously published method^40^. IOP monitoring with Tonopen (Reichert Technologies, Depew, NY) and topical postoperative treatment with 0.5% Timolol (Akorn), 0.3% Ofloxacin (Akorn) and triple antibiotic ointment (Akorn), were performed for 3 days after gas displacement and after cell injection. Rabbits were examined using indirect ophthalmoscopy after cell injection, 1 week, 2 weeks and 4 weeks. For fundus and OCT imaging, a spectral-domain OCT Spectralis (Heidelberg Engineering, Heidelberg, Germany) was used immediately after cell injection, at 2 and 4 weeks of follow-up. A PVR score grading system was developed to assess severity of disease. The score was determined by combination of the most severe phenotypes identified by indirect ophthalmoscopy, fundus imaging and OCT. Representative images of each severity stage identified are showed in Supplementary Fig. S10. Under deep anesthesia, animals were euthanized and eyes collected after 14 or 28 days of follow-up by injection of pentobarbital (120 mg/kg) (Fatal Plus, Vortech, Dearborn, MI).

### Ocular toxicity assessment

To evaluate anterior segment safety and tolerability of the topical application of a nanoemulsion, the SPOTS system, based on the McDonald-Shadduck and Hackett-McDonald scales, was followed^75^. For posterior segment toxicity assessment, TUNEL (In Situ Cell Death Detection Kit, TMR red, 12156792910, Roche, Indianapolis-Marion County, IN) and morphology comparison by H&E staining were performed in retinal tissue sections. Also, IOPs were measured before any procedure (baseline) and after 2 and 4 weeks of follow-up.

### ERG recording

To test potential non-lethal cell dysfunction in retinal tissue we performed ERG analysis in C57BL6/J mice. Animals were distributed into three groups: Untreated, vehicle and eNano-Ro5. For those receiving topical nanoemulsion, 1 drop was applied 4 times a day for 7 days and then ERG recording protocol was done. Following overnight dark adaptation, the animals were prepared for ERG recording under dim red light. While under anesthesia with a mixture of Ketamine and Xylazine, the animal body temperature was maintained at 38 °C, using a warm heating blanket, and their pupils were dilated using a drop of 1% Tropicamide applied on the corneal surface. One drop of Genteal was applied to the cornea of the untreated eye to prevent dehydration. A drop of 0.9% sterile saline was applied on the cornea of the treated eye to prevent dehydration and to allow electrical contact with the recording electrode. A 25-gauge platinum needle, inserted subcutaneously in the forehead, served as reference electrode, while a needle inserted subcutaneously near the tail served as the ground electrode. A series of flash intensities were produced by a Ganzfeld controlled by the Diagnosys Espion3 to test both scotopic and photopic responses.

### Histopathological evaluation of C-PVR injected eyes

After euthanasia, eyes were enucleated and submerged in Davidson’s fixative (Millipore Sigma). After 24 h at RT, a small 1 × 1 mm scleral window was performed next to the corneal limbus to facilitate fixative penetration within the eye and submerged again in fixative for another 24 h at RT. Then, samples were transferred to 70% ethanol for at least 24 h and 5 μm serial sections were obtained by standard paraffin embedding procedure. Hematoxylin and eosin staining was used for morphologic studies of ocular tissues. Also, to confirm presence of human cells within rabbit vitreous cavity a specific antibody against human antigen was used: rat anti-HLA Class I Xenograft marker antibody (1:200; SM2012P Acris, OriGene Technologies, Inc., Rockville, MD). Mouse anti-PCNA antibody (1:100; ab29, Abcam, Cambridge, UK) was used to detect the presence of proliferating cells within the pathological tissues and mouse anti-fibronectin (1:500, FBN11, Invitrogen, Carlsbad, CA) antibody, a PVR marker, was also performed. Slides were washed with PBS and permeabilized with 0.5% Triton X-100 in PBS for 5 min and blocked (10% goat serum in PBS) for 1 h at RT. The primary antibody was prepared in antibody dilution buffer (5% goat serum) and incubated overnight at 4 °C. Slides were washed with PBS and incubated with goat anti-rat and goat anti-mouse Alexa Fluor 594 secondary antibody, respectively (1:300; A-11007, A-11005, Life Technologies) for 2 h at RT. Slides were mounted and visualized using Prolong Gold Antifade Reagent with DAPI (P36935, Invitrogen). Images were obtained using an EVOS FL automated stage live cell imaging system (Life Technologies).

### Nanoemulsion (eNano-Ro5) preparation

Lecithin was extracted in sterile conditions from commercially available capsules and mixed with isopropyl myristate (172472, Millipore Sigma) in a 1:1 proportion as surfactant and organic phase, respectively. Then, 10 mg of Ro5-3335 (219506, Millipore Sigma) were added to the mixture to develop eNano-Ro5. Sterile PBS was added to reach a final concentration of 7.92 mM. The mixture was homogenized at 4,000 rpm for 6 min with a PT10-35 GT Kinematica Polytron homogenizer (Kinematica AG, Luzern, Switzerland) and subjected to sonication with a Qsonica XL-2000 sonicator (Qsonica LLC, Newtown, CT) at 15 kW of power for 10 min in ice to avoid overheating of the product. For the vehicle formulation used as a control, the same protocol was followed without adding Ro5-3335 to the mixture.

### eNano-Ro5 characterization

Particle size was characterized by DLS equipment with a DynaPro NanoStar (Wyatt Technology, Santa Barbara, CA) with an operation angle of 90°. The data were collected in a series of ten measurements from duplicate at 25 °C. In order to evaluate stability of the nanoemulsion formulation, changes in nanodrop size were evaluated by DLS at different time points. To quantify the amount of Ro5-3335 released from the nanoemulsion, eNano-Ro5 was dispersed in 1 mL of PBS (pH 7.4) to a final concentration of 0.021 mg/ml. The mixture (1 mL) was placed into a Float-A-Lyzer G2 Dialysis Device (3.5–5 kD) (1210W03, Thomas Scientific, Swedesboro, NJ) and free dialyzed in 200 mL of PBS, to allow sink conditions. The system was kept at 37 °C and 150 strokes. At regular time intervals, aliquots of the release medium were withdrawn and replaced with an equal volume of fresh buffer. Finally, the concentration of Ro5-3335 was measured by LC–MS/MS.

### Quantification of Ro5-3335 by liquid chromatography–tandem mass spectrometry in rabbit ocular tissues

Samples from the cornea, aqueous humor and vitreous were collected after 2 and 4 weeks of treatment. Corneal tissue was thinly ground using a scalpel blade, homogenized in 0.5 mL of Tris-buffered saline (TBS) (pH 9.0, 0.5 M) for 10 s intervals with a PT10-35 GT Kinematica Polytron homogenizer (Kinematica AG) and then subjected to sonication (10 s, 0.5 kW) with a Qsonica XL-2000 sonicator (Qsonica LLC). For the aqueous humor samples, a volume of 100 μL was collected and mixed with 0.5 mL of TBS buffer. Vitreous samples were homogenized for 10 s and 0.5 ml of the mixture was taken before being combined with 0.5 mL of TBS buffer. Subsequently, 0.5 mL of n-butyl chloride was added to each of the cornea, aqueous humor and vitreous samples and incubated at 4 °C for 25 min to allow for mass transfer. Samples were centrifuged for 5 min at 12,000 rpm and the organic phase was collected. This extraction procedure was repeated three times. At the final stage, eluates were dried and reconstituted in 50 μL of dimethyl sulfoxide (DMSO). To verify the extraction efficiency of the method, aliquots of cornea, aqueous humor and vitreous samples were spiked with 50 ng/μL prior extraction. Successively, samples were prepared according to the protocol described above. Non-spiked tissues served as blanks (non-extracted samples). The recovery was analyzed by comparing the response of extracted and non-extracted samples (Fig. 4e). LC–MS/MS was used to quantify Ro5-3335 on each sample. Calibration curves of Ro5-3335 were prepared by serial dilutions in DMSO to produce concentrations between the range of 0.1 to 50 pg/μL. The compounds were separated on an Allure PFPP column (2.1 × 150 mm, 5 μm) using a 6460 LC–MS/MS system (Agilent, Santa Clara, CA). The mobile phase used was 0.1% formic acid in acetonitrile, and the sample injection volume was 10 μL. A constant flow rate of 0.5 mL/min was utilized with a gradient elution method over 10 min to operate the liquid chromatograph. The mass spectrometer portion of the LC–MS/MS was set at a temperature of 350 °C with a gas flow of 12 L/min, sheath gas temperature 400 °C, sheath gas flow 12 L/min, capillary voltage 3.5 kV and nebulizer 35 psi. The electrospray ionization positive ion mode provided the maximum ionization and optimized retention time of the Ro5-3335 (5.17 min).

### Statistical analyses

Data are shown as mean ± SEM or median. For statistical analysis, two-tailed unpaired Mann–Whitney test, unpaired T-test, one-way ANOVA or two-way ANOVA were used. NS, not significant, *p < 0.05, **p ≤ 0.01, ***p ≤ 0.001 and ****p ≤ 0.0001. Sample size was calculated by power calculation according to previous experiments.

## Supplementary information

Supplementary Information.

## Data Availability

All data associated with this study are present in the paper or the Supplementary Materials.

## References

[1] Weichel ED, Bower KS, Colyer MH (2010). Chorioretinectomy for perforating or severe intraocular foreign body injuries. Graefes Arch. Clin. Exp. Ophthalmol..

[2] Martini B (1992). Proliferative vitreo-retinal disorders: Experimental models in vivo and in vitro. Acta Ophthalmol. Suppl..

[3] Colyer MH, Chun DW, Bower KS, Dick JS, Weichel ED (2008). Perforating globe injuries during operation Iraqi Freedom. Ophthalmology.

[4] Pastor JC, de la Rua ER, Martin F (2002). Proliferative vitreoretinopathy: Risk factors and pathobiology. Prog. Retin. Eye Res..

[5] Eliott D, Stryjewski TP, Andreoli MT, Andreoli CM (2017). Smoking is a risk factor for proliferative vitreoretinopathy after traumatic retinal detachment. Retina (Philadelphia, Pa.).

[6] Ryan SJ (1993). Traction retinal detachment. XLIX Edward Jackson Memorial Lecture. Am. J. Ophthalmol..

[7] Pastor JC (1998). Proliferative vitreoretinopathy: An overview. Surv. Ophthalmol..

[8] Cheema RA (2007). Triamcinolone acetonide as an adjuvant in the surgical treatment of retinal detachment with proliferative vitreoretinopathy. Ophthalmic Surg. Lasers Imaging.

[9] Ahmadieh H (2008). Triamcinolone acetonide in silicone-filled eyes as adjunctive treatment for proliferative vitreoretinopathy: A randomized clinical trial. Ophthalmology.

[10] Reibaldi M (2013). Rhegmatogenous retinal detachment with a high risk of proliferative vitreoretinopathy treated with episcleral surgery and an intravitreal dexamethasone 0.7-mg implant. Case Rep. Ophthalmol..

[11] Blumenkranz M, Hernandez E, Ophir A, Norton EW (1984). 5-fluorouracil: New applications in complicated retinal detachment for an established antimetabolite. Ophthalmology.

[12] Asaria RH (2001). Adjuvant 5-fluorouracil and heparin prevents proliferative vitreoretinopathy: Results from a randomized, double-blind, controlled clinical trial. Ophthalmology.

[13] Charteris DG (2004). A randomized controlled trial of combined 5-fluorouracil and low-molecular-weight heparin in management of established proliferative vitreoretinopathy. Ophthalmology.

[14] Chang YC, Hu DN, Wu WC (2008). Effect of oral 13-cis-retinoic acid treatment on postoperative clinical outcome of eyes with proliferative vitreoretinopathy. Am. J. Ophthalmol..

[15] Hou H (2015). A novel approach of daunorubicin application on formation of proliferative retinopathy using a porous silicon controlled delivery system: pharmacodynamics. Invest. Ophthalmol. Vis. Sci..

[16] Hsu J (2015). Effect of serial intrasilicone oil bevacizumab injections in eyes with recurrent proliferative vitreoretinopathy retinal detachment. Am. J. Ophthalmol..

[17] Charteris DG (1995). Proliferative vitreoretinopathy: Pathobiology, surgical management, and adjunctive treatment. Br. J. Ophthalmol..

[18] Khan MA, Brady CJ, Kaiser RS (2015). Clinical management of proliferative vitreoretinopathy: an update. Retina (Philadelphia, Pa.).

[19] Lamouille S, Xu J, Derynck R (2014). Molecular mechanisms of epithelial-mesenchymal transition. Nat. Rev. Mol. Cell Biol..

[20] Kalluri R, Weinberg RA (2009). The basics of epithelial-mesenchymal transition. J. Clin. Investig..

[21] Pastor JC (2016). Proliferative vitreoretinopathy: A new concept of disease pathogenesis and practical consequences. Prog. Retin. Eye Res..

[22] Chiba C (2014). The retinal pigment epithelium: an important player of retinal disorders and regeneration. Exp. Eye Res..

[23] Shu DY, Lovicu FJ (2017). Myofibroblast transdifferentiation: The dark force in ocular wound healing and fibrosis. Prog. Retin. Eye Res..

[24] Pennock S, Haddock LJ, Eliott D, Mukai S, Kazlauskas A (2014). Is neutralizing vitreal growth factors a viable strategy to prevent proliferative vitreoretinopathy?. Prog. Retin. Eye Res..

[25] Morescalchi F (2013). Proliferative vitreoretinopathy after eye injuries: an overexpression of growth factors and cytokines leading to a retinal keloid. Mediators Inflamm..

[26] Nakagawa M, Refojo MF, Marin JF, Doi M, Tolentino FI (1995). Retinoic acid in silicone and silicone-fluorosilicone copolymer oils in a rabbit model of proliferative vitreoretinopathy. Invest. Ophthalmol. Vis. Sci..

[27] Andrews A (1999). Platelet-derived growth factor plays a key role in proliferative vitreoretinopathy. Invest. Ophthalmol. Vis. Sci..

[28] Radtke ND, Tano Y, Chandler D, Machemer R (1981). Simulation of massive periretinal proliferation by autotransplantation of retinal pigment epithelial cells in rabbits. Am. J. Ophthalmol..

[29] Zhao HM, Sheng MJ, Yu J (2014). Expression of IGFBP-6 in a proliferative vitreoretinopathy rat model and its effects on retinal pigment epithelial cell proliferation and migration. Int. J. Ophthalmol..

[30] Cleary PE, Ryan SJ (1979). Experimental posterior penetrating eye injury in the rabbit. I. Method of production and natural history. Br. J. Ophthalmol..

[31] Bochaton-Piallat ML (2000). TGF-beta1, TGF-beta receptor II and ED-A fibronectin expression in myofibroblast of vitreoretinopathy. Invest. Ophthalmol. Vis. Sci..

[32] Rouberol F, Chiquet C (2014). Proliferative vitreoretinopathy: pathophysiology and clinical diagnosis. J. Fr. Ophtalmol..

[33] Agrawal RN (2007). In vivo models of proliferative vitreoretinopathy. Nat. Protoc..

[34] Fisher SK, Lewis GP (2003). Muller cell and neuronal remodeling in retinal detachment and reattachment and their potential consequences for visual recovery: A review and reconsideration of recent data. Vision Res..

[35] Iandiev I (2006). Glial cell reactivity in a porcine model of retinal detachment. Invest. Ophthalmol. Vis. Sci..

[36] Kirchhof B, Sorgente N (1989). Pathogenesis of proliferative vitreoretinopathy. Modulation of retinal pigment epithelial cell functions by vitreous and macrophages. Dev. Ophthalmol..

[37] Nagasaki H, Shinagawa K, Mochizuki M (1998). Risk factors for proliferative vitreoretinopathy. Prog. Retin. Eye. Res..

[38] Garweg JG, Tappeiner C, Halberstadt M (2013). Pathophysiology of proliferative vitreoretinopathy in retinal detachment. Surv. Ophthalmol..

[39] Moysidis SN, Thanos A, Vavvas DG (2012). Mechanisms of inflammation in proliferative vitreoretinopathy: from bench to bedside. Mediators Inflamm..

[40] Amarnani D (2017). Effect of methotrexate on an in vitro patient-derived model of proliferative vitreoretinopathy. Invest. Ophthalmol. Vis. Sci..

[41] Lam JD (2017). Identification of RUNX1 as a mediator of aberrant retinal angiogenesis. Diabetes.

[42] Illendula A (2016). Small molecule inhibitor of CBFbeta-RUNX binding for RUNX transcription factor driven cancers. EBioMedicine..

[43] Bartfeld D (2002). DNA recognition by the RUNX1 transcription factor is mediated by an allosteric transition in the RUNT domain and by DNA bending. Structure.

[44] Bellissimo DC, Speck NA (2017). RUNX1 mutations in Inherited and sporadic leukemia. Front. Cell Dev. Biol..

[45] Keita M (2013). The RUNX1 transcription factor is expressed in serous epithelial ovarian carcinoma and contributes to cell proliferation, migration and invasion. Cell Cycle.

[46] Hong D (2017). Runx1 stabilizes the mammary epithelial cell phenotype and prevents epithelial to mesenchymal transition. Oncotarget..

[47] Zhou T (2018). Runt-related transcription factor 1 (RUNX1) promotes TGF-β-induced renal tubular epithelial-to-mesenchymal transition (EMT) and renal fibrosis through the PI3K subunit p110δ. EBioMedicine.

[48] Zhao K (2019). RUNX1 contributes to the mesenchymal subtype of glioblastoma in a TGFbeta pathway-dependent manner. Cell Death Dis..

[49] Chen MJ, Yokomizo T, Zeigler BM, Dzierzak E, Speck NA (2009). Runx1 is required for the endothelial to haematopoietic cell transition but not thereafter. Nature.

[50] Richard C (2013). Endothelio-mesenchymal interaction controls runx1 expression and modulates the notch pathway to initiate aortic hematopoiesis. Dev. Cell..

[51] Li Q (2019). RUNX1 promotes tumour metastasis by activating the Wnt/beta-catenin signalling pathway and EMT in colorectal cancer. J. Exp. Clin. Cancer Res..

[52] VanOudenhove JJ (2016). Transient RUNX1 expression during early mesendodermal differentiation of hESCs promotes epithelial to mesenchymal transition through TGFB2 signaling. Stem Cell Rep.

[53] Mercado-Matos J, Matthew-Onabanjo AN, Shaw LM (2017). RUNX1 and breast cancer. Oncotarget.

[54] Cunningham L (2012). Identification of benzodiazepine Ro5-3335 as an inhibitor of CBF leukemia through quantitative high throughput screen against RUNX1-CBFbeta interaction. Proc. Natl. Acad. Sci..

[55] Guenther SR (2019). Comparison of surgically excised premacular membranes in eyes with macular pucker and proliferative vitreoretinopathy. Curr. Eye Res..

[56] Oberstein SY (2011). Cell proliferation in human epiretinal membranes: characterization of cell types and correlation with disease condition and duration. Mol. Vis..

[57] Feist RM, King JL, Morris R, Witherspoon CD, Guidry C (2014). Myofibroblast and extracellular matrix origins in proliferative vitreoretinopathy. Graefes Arch. Clin. Exp. Ophthalmol..

[58] Fastenberg DM, Diddie KR, Sorgente N, Ryan SJ (1982). A comparison of different cellular inocula in an experimental model of massive periretinal proliferation. Am. J. Ophthalmol..

[59] Vandamme TF (2002). Microemulsions as ocular drug delivery systems: recent developments and future challenges. Prog. Retin. Eye Res..

[60] Hou H, Nudleman E, Weinreb RN (2018). Animal models of proliferative vitreoretinopathy and their use in pharmaceutical investigations. Ophthalmic Res..

[61] Del Amo EM (2017). Pharmacokinetic aspects of retinal drug delivery. Prog. Retin. Eye Res..

[62] Del Amo EM, Urtti A (2015). Rabbit as an animal model for intravitreal pharmacokinetics: Clinical predictability and quality of the published data. Exp. Eye Res..

[63] Grzybowski A (2018). 2018 Update on intravitreal injections: Euretina expert consensus recommendations. Ophthalmologica..

[64] Falavarjani KG, Nguyen QD (2013). Adverse events and complications associated with intravitreal injection of anti-VEGF agents: A review of literature. Eye (London, England)..

[65] Higashide T, Murotani E, Saito Y, Ohkubo S, Sugiyama K (2012). Adverse events associated with intraocular injections of bevacizumab in eyes with neovascular glaucoma. Graefes Arch. Clin. Exp. Ophthalmol..

[66] Xu K (2018). Endophthalmitis after intravitreal injection of vascular endothelial growth factor inhibitors: Management and visual outcomes. Ophthalmology.

[67] Haller JA (2013). Current anti-vascular endothelial growth factor dosing regimens: benefits and burden. Ophthalmology.

[68] Moore HM (2011). Biospecimen reporting for improved study quality (BRISQ). Cancer Cytopathol..

[69] Fragiadaki M, Mason RM (2011). Epithelial-mesenchymal transition in renal fibrosis - evidence for and against. Int. J. Exp. Pathol..

[70] Mitsuda Y (2018). RUNX1 positively regulates the ErbB2/HER2 signaling pathway through modulating SOS1 expression in gastric cancer cells. Sci. Rep..

[71] Wang CH (2019). Dieckol inhibits non-small-cell lung cancer cell proliferation and migration by regulating the PI3K/AKT signaling pathway. J. Biochem. Mol. Toxicol..

[72] Chen J, Qin R (2020). MicroRNA-138-5p regulates the development of spinal cord injury by targeting SIRT1. Mol. Med. Rep..

[73] Kern S, Feng HZ, Wei H, Cala S, Jin JP (2013). Up-regulation of alpha-smooth muscle actin in cardiomyocytes from non-hypertrophic and non-failing transgenic mouse hearts expressing N-terminal truncated cardiac troponin I. FEBS Open Bio..

[74] Kilkenny C, Browne WJ, Cuthill IC, Emerson M, Altman DG (2010). Improving bioscience research reporting: the ARRIVE guidelines for reporting animal research. PLoS Biol..

[75] Eaton JS, Miller PE, Bentley E, Thomasy SM, Murphy CJ (2017). The SPOTS system: An ocular scoring system optimized for use in modern preclinical drug development and toxicology. J. Ocul. Pharmacol. Ther..

