# Supplementary material for: Topical delivery of a small molecule RUNX1 transcription factor inhibitor for the treatment of proliferative vitreoretinopathy
Source: Sci Rep. 2020 Nov 30;10:20554. doi: 10.1038/s41598-020-77254-0 (PMC7705016; doi:10.1038/s41598-020-77254-0)
Supplement: Supplementary file 1 — Supplementary Information. [file 41598_2020_77254_MOESM1_ESM.docx]

Supplementary Material

Topical delivery of a small molecule RUNX1 transcription factor inhibitor for the treatment of proliferative vitreoretinopathy

Santiago Delgado-Tirado ^1^†, Dhanesh Amarnani ^1^†, Guannan Zhao ^1^, Elizabeth Rossin ^2^, Dean Eliott ^2^, John B. Miller ^2^, Whitney A. Greene ^3^, Leslie Ramos ^1^, Said Arevalo-Alquichire ^1,4^, David Leyton-Cifuentes ^1,5^, Lucia Gonzalez-Buendia ^1^, Daniela Isaacs-Bernal ^1,4^, Hannah Whitmore ^1^, Natalia Chmielewska ^1,6^, Brandon V. Duffy ^1,7^, Eric Kim ^1^, Heuy-Ching Wang ^3^, Jose M. Ruiz-Moreno ^8^, Leo A. Kim ^1,2^*, Joseph F. Arboleda-Velasquez ^1,5^*

Supplementary Figure S1


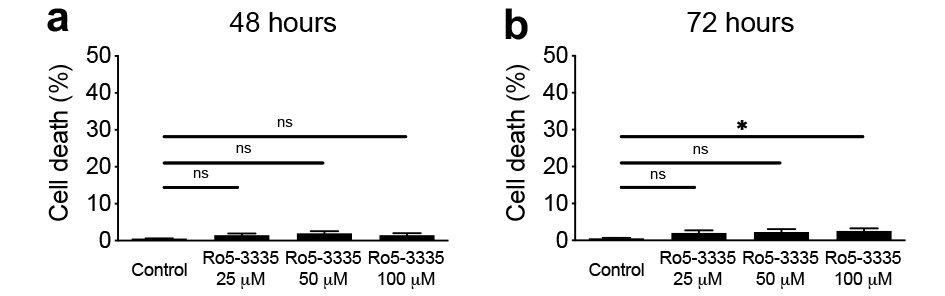


**Supplementary Figure S1.** LDH cytotoxicity assay of C-PVR cells treated with different doses of RUNX1 inhibitor. Low levels of LDH were detected in these samples at 48 **(a)** and 72 hours **(b)**. *p<0.01, one-way ANOVA; n=4 represented as mean ± SEM.

**
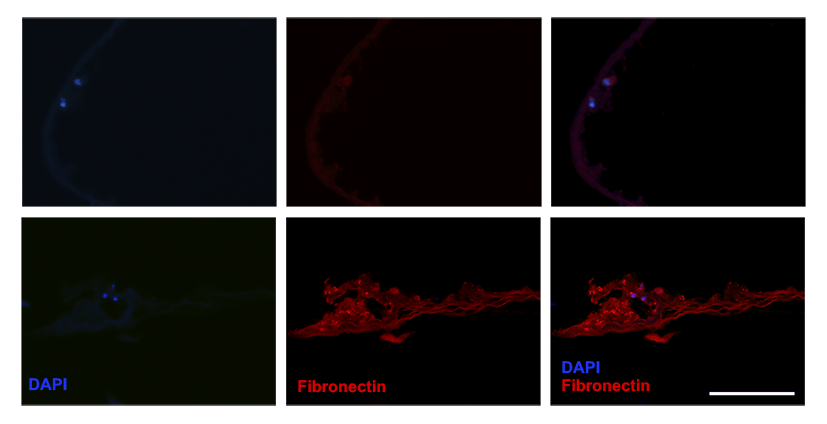
**Supplementary Figure S2

**Supplementary figure S2.** Experimental PVR membrane staining showing intense positivity for Fibronectin, a marker of early PVR (bottom panel). Top panel showing negative control. Scale bar: 100 μm.


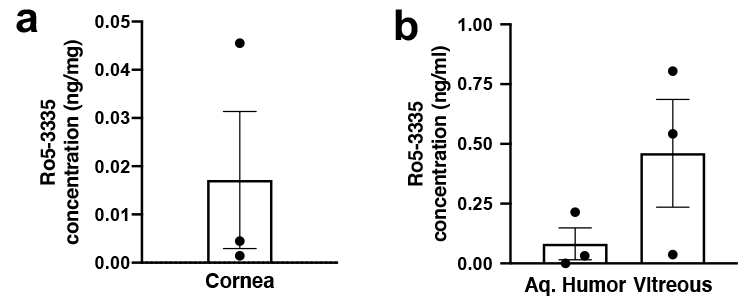
Supplementary Figure S3

**Supplementary figure S3.** Ro5-3335 concentration in rabbit cornea **(a)**, aqueous humor and vitreous **(b)** after 2 weeks of treatment with topical eNano-Ro5 by LC-MS/MS. Results are expressed as mean ± SEM, n=3.

Supplementary Figure S4

**Supplementary figure S4.** Ocular toxicity results after topical treatment with eNano-Ro5. **(a)** No pathological anterior segment findings were identified after 2 or 4 weeks of follow-up. **(b)** No changes in IOP at any evaluated timepoint were found (Normal range: 10-25 mmHg), (two-way ANOVA, represented as mean ± SEM). **(c)** Anterior segment toxicity score results obtained following the Semiquantitative Preclinical Ocular Toxicology Scoring System (SPOTS) guidelines are shown. Treatments were applied topically three times a day during 4 weeks. ns: not significant PLR: Pupillary light reflex. **(d)** No changes in a- or b-wave amplitudes for scotopic and photopic responses in comparison between treatment groups.


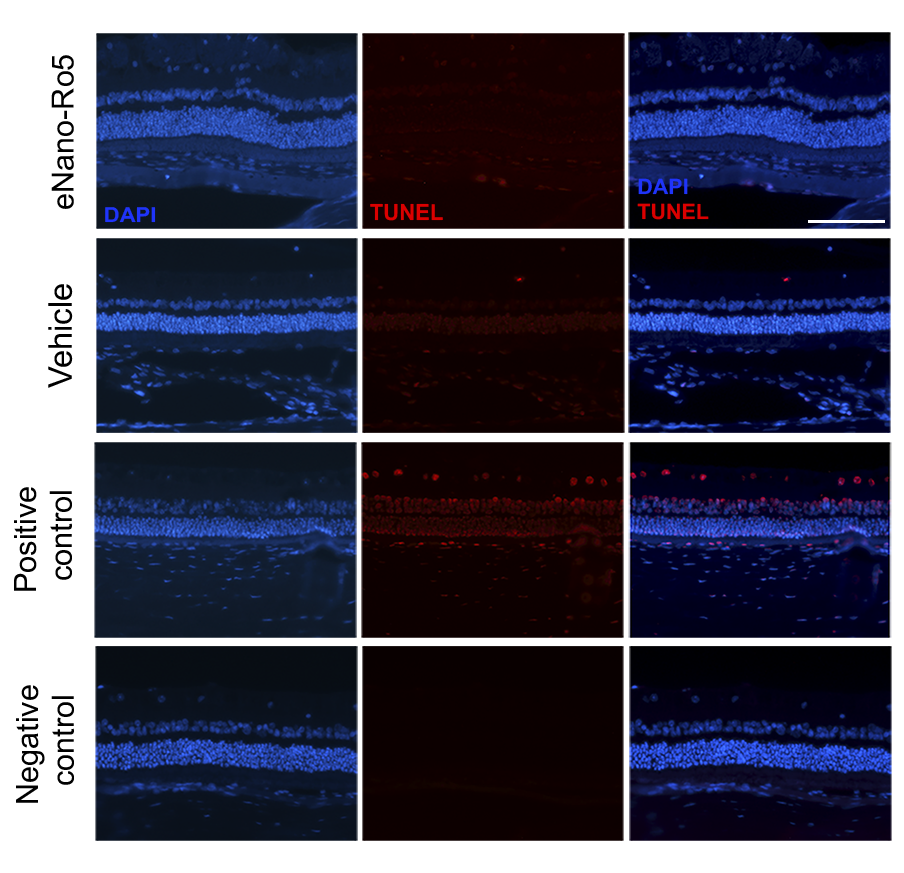
Supplementary Figure S5

**Supplementary figure S5.** Representative images of TUNEL staining in C-PVR injected eyes. There are no significant differences observed between study groups. Scale bar: 100 μm.

Supplementary Figure S6

**
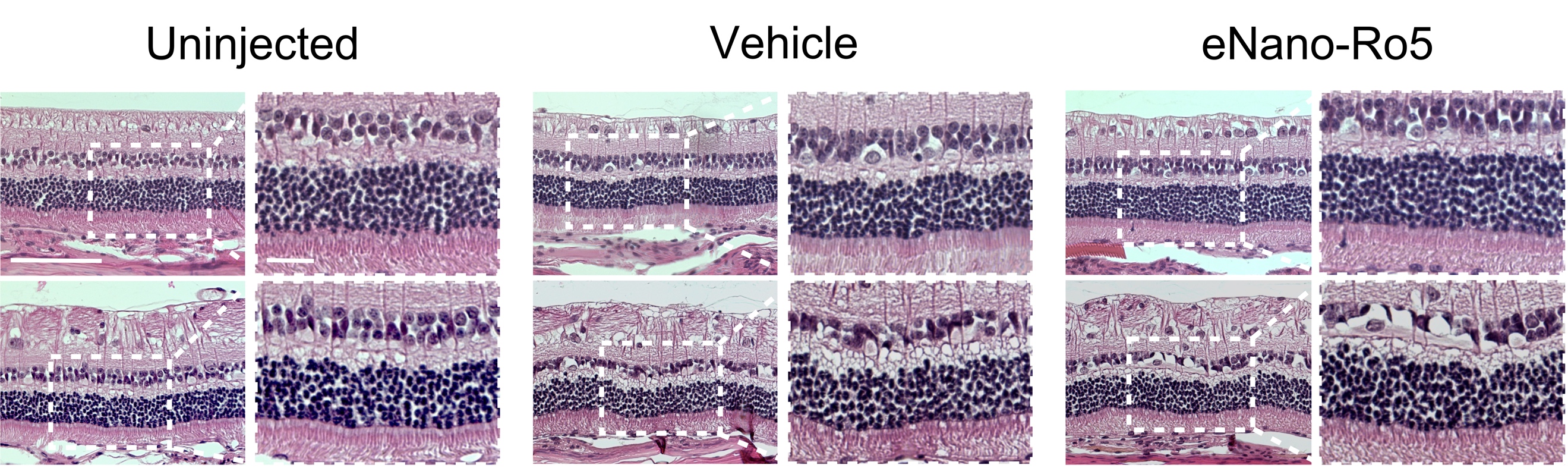
**

**Supplementary figure S6.** Representative images of rabbit retinal tissue stained with H&E. Uninjected left eye where no procedures have been performed is showed as control. Similar histology changes in neuroretina are observed after PVR model induction in both treatment groups. No overt signs of toxicity are observed. Insets depict magnified areas. Scale bars: 100μm and 25μm.

Supplementary Figure S7


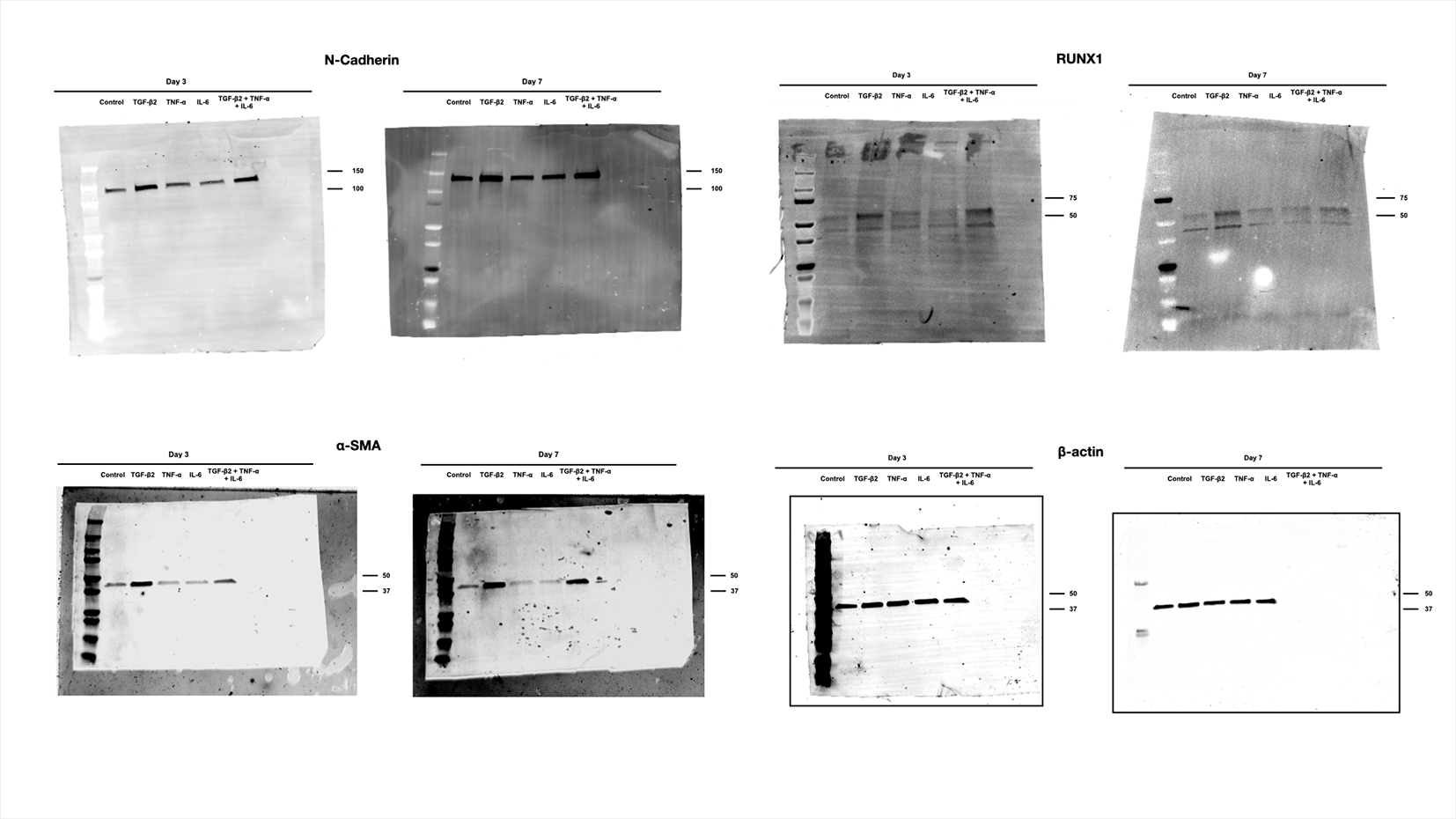

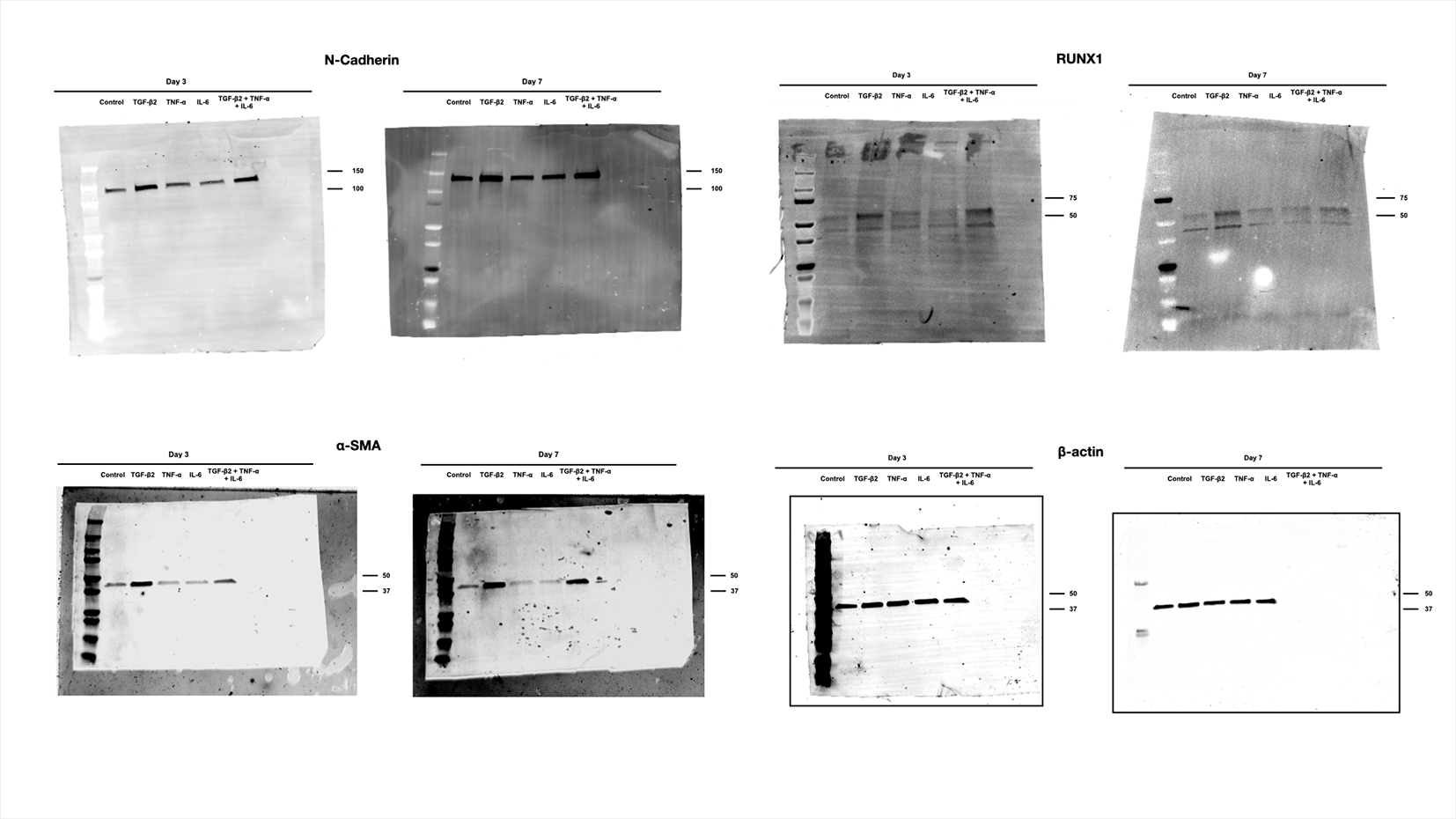


**Supplementary figure S7.** Original full length immunoblots of images presented in Fig. 2c


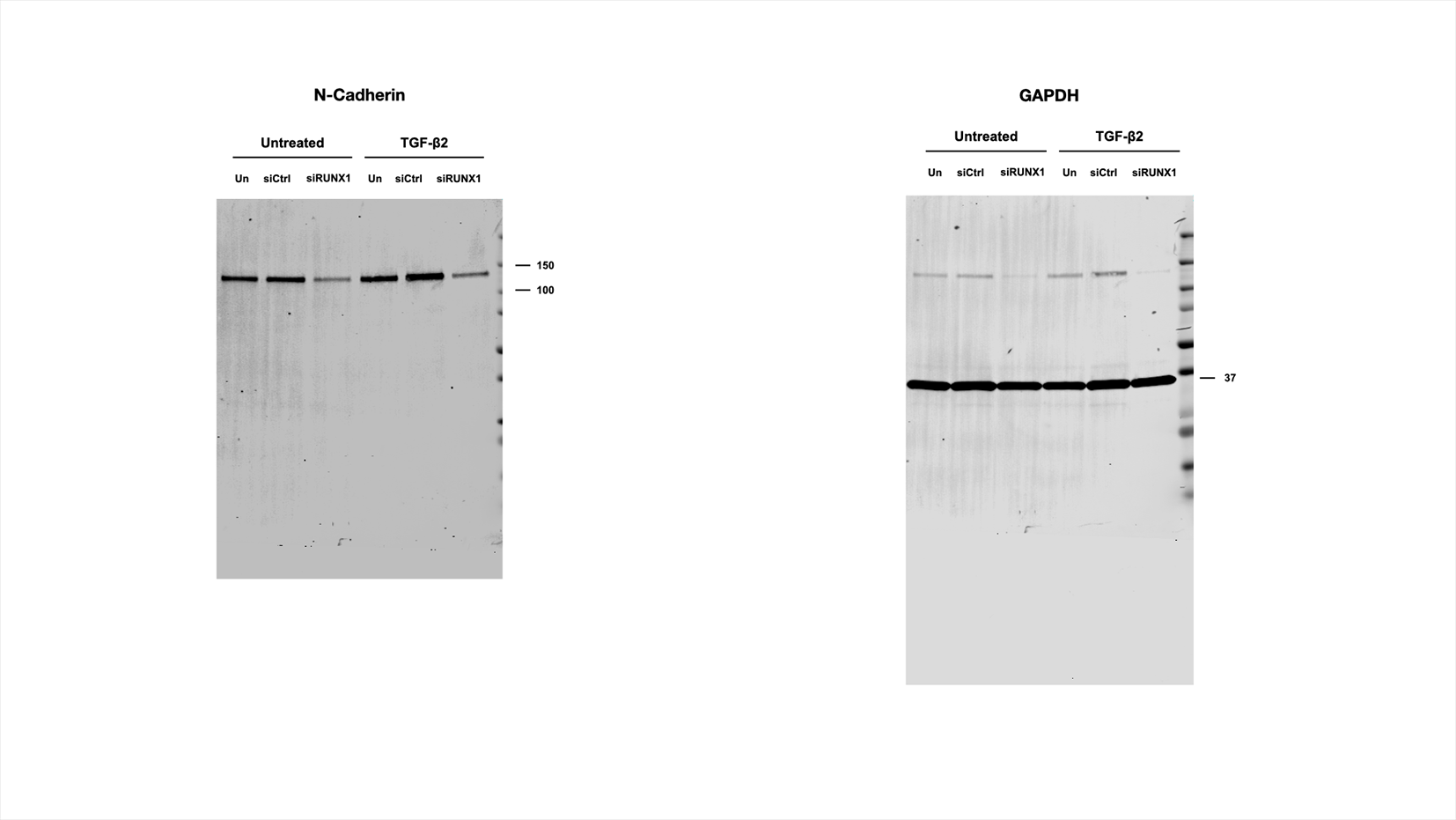
Supplementary Figure S8

**Supplementary figure S8**. Original full length immunoblots of images presented in Fig. 2e


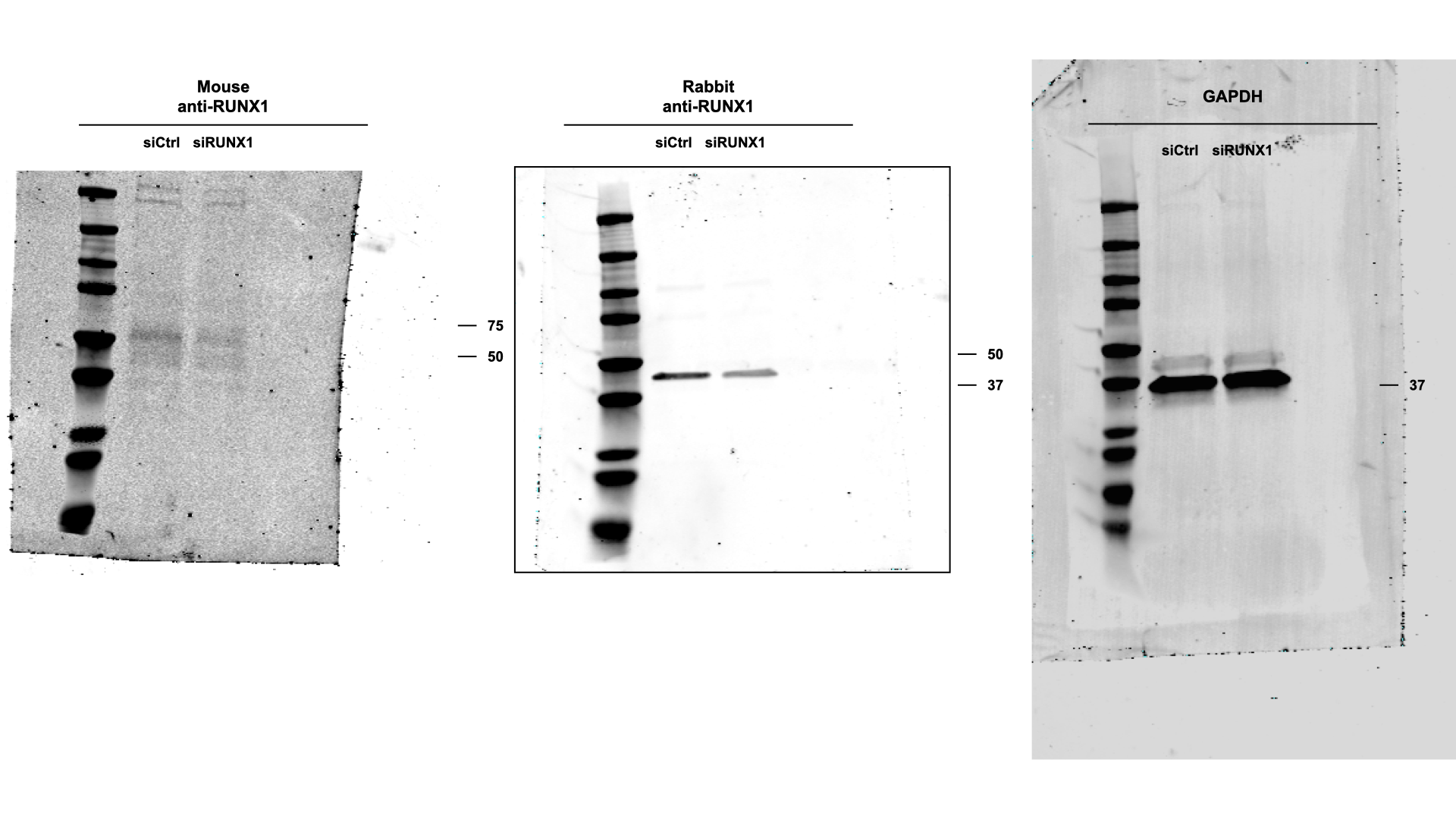
Supplementary Figure S9

**Supplementary figure S9.** Original full length immunoblots of images presented in Fig. 2h


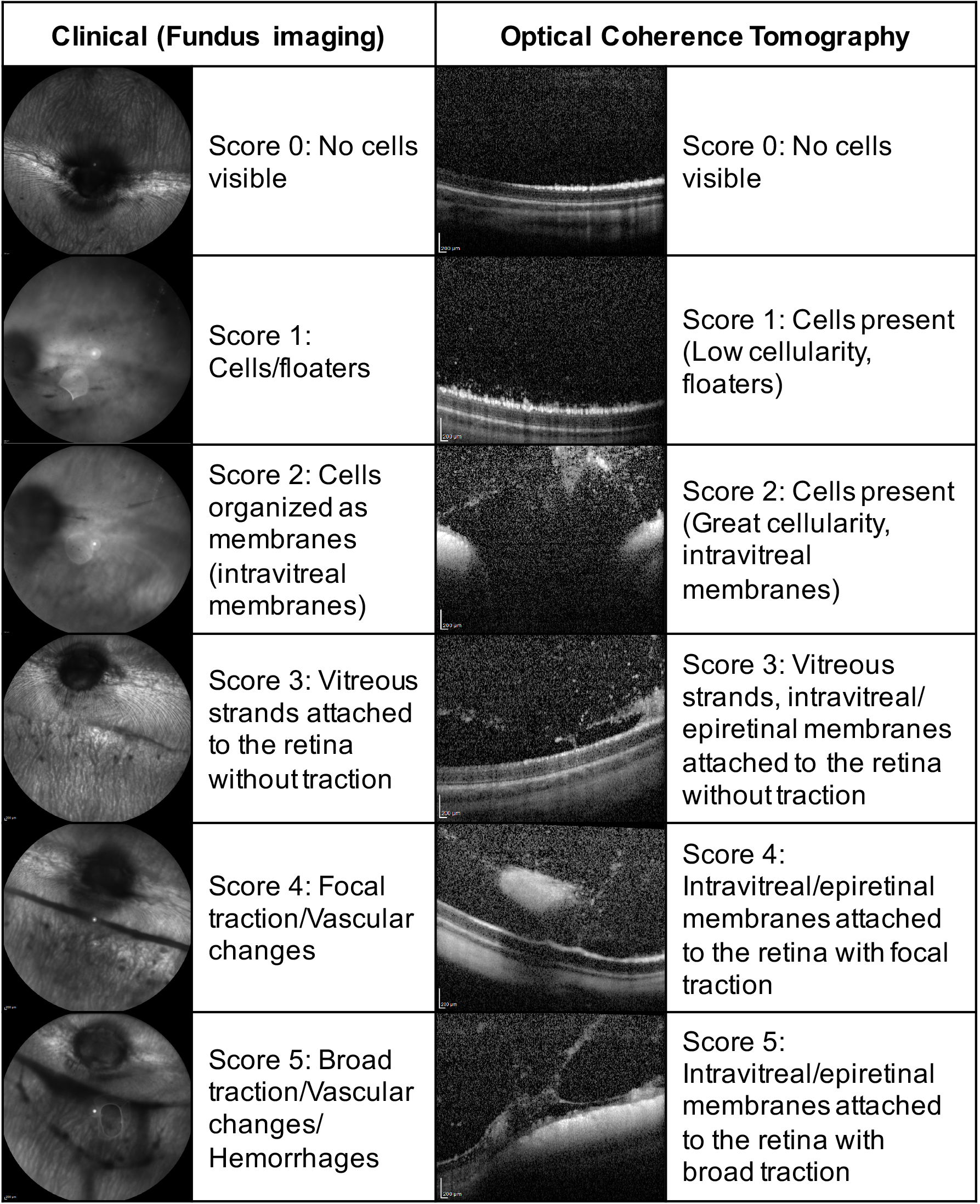
Supplementary Figure S10

**Supplementary figure S10.** Representative images showing each severity stage identified in experimental PVR.

Supplementary Table S1. PVR Score grading system.

| **Table S1.** PVR Score grading system | | | |
| --- | --- | --- | --- |
| **Score** | **Clinical (Ophthalmoscopy + Fundus images)** | **Score** | **Optical Coherence Tomography** |
| 0 | No cells visible | 0 | No cells visible |
| 1 | Cells/floaters | 1 | Cells present (Low cellularity, floaters) |
| 2 | Cells organized as membranes (intravitreal membranes) | 2 | Cells present (Great cellularity, intravitreal membranes) |
| 3 | Vitreous strands attached to the retina without traction | 3 | Vitreous strands, intravitreal/epiretinal membranes attached to the retina without traction |
| 4 | Focal traction/Vascular changes | 4 | Intravitreal/Epiretinal membranes attached to the retina with focal traction |
| 5 | Broad traction/Vascular changes/Hemorrhages | 5 | Intravitreal/Epiretinal membranes attached to the retina with broad traction |
| 6 | Focal retinal detachment | 6 | Focal retinal detachment |
| 7 | Extensive retinal detachment/Holes/Tears | 7 | Extensive retinal detachment/Holes/Tears |
| +1 | Presence of retrolental proliferation was scored with one extra point of severity. | | |
